# Quinoline based thiosemicarbazones as colorimetric chemosensors for fluoride and cyanide ions and DFT studies

**DOI:** 10.1038/s41598-022-08860-3

**Published:** 2022-03-23

**Authors:** Rabia Basri, Nadeem Ahmed, Muhammad Khalid, Muhammad Usman Khan, Muhammad Abdullah, Asad Syed, Abdallah M. Elgorban, Salim S. Al-Rejaie, Ataualpa Albert Carmo Braga, Zahid Shafiq

**Affiliations:** 1 Institute of Chemical Sciences, Bahauddin Zakariya University, Multan, 60800 Pakistan; 2 Department of Chemistry, Khwaja Fareed University of Engineering & Information Technology, Rahim Yar Khan, 64200 Pakistan; 3 Department of Chemistry, University of Okara, Okara, 56300 Pakistan; 4 Department of Chemistry, Colorado State University, Fort Collins, CO 80523 USA; 5 Department of Botany and Microbiology, College of Science, King Saud University, P.O. 2455, Riyadh, 11451 Saudi Arabia; 6 Department of Pharmacology and Toxicology, College of Pharmacy, King Saud University, P.O. Box 55760, Riyadh, 11451 Saudi Arabia; 7 Departamento de Química Fundamental, Instituto de Química, Universidade de São Paulo, Av. Prof. Lineu Prestes, 748, São Paulo, 05508-000 Brazil

**Keywords:** Organic chemistry, Theoretical chemistry, Sensors, Chemistry, Analytical chemistry

## Abstract

High toxicity and extensive accessibility of fluoride and cyanide ions in diverse environmental media encouraged attention for scheming well-organized probes for their detection. Keeping in mind we have designed and synthesized thiosemicarbazone-based chemosensors RB-1, RB-2 and RB-3 for the detection of fluoride and cyanide ions. The structural elucidation of the synthesized chemosensors is done by employing different analytical techniques including nuclear magnetic resonance and electronic absorption specrtoscopies. Admirable detection limit, binding constant and fast response time (2 s) to F^−^ and CN^−^ ions enlarged the applications of these chemosensors. Additional confirmation of the sensing ability of these chemosensors is derived from DFT and TDDFT calculations with M06/6-311G(d,p) method by performing FMO, UV–Vis, QTAIM and global reactivity parameters elucidation. Overall results point out that investigated chemosensors are suitable candidates for sensing the F^−^ ions. These chemosensors were successfully applied to detect F^−^ ions in a commercial toothpaste sample.

## Introduction

In supramolecular chemistry, selective and sensitive detection of anions have attracted a great attention due to their biomedical, environmental and chemical applications^1–5^. Among the numerous anions CN^−^ and F^−^ are immensely harmful to environment and human health^6^. The fluoride is the smallest and most electronegative ion that promotes healthy bone growth and avoids dental cavities due to which it is considered as a micronutrient^7^. The F^−^ ions are commonly present in toothpaste, pharmaceutical agents and drinking water due to their significant role in avoiding dental caries and treatment of osteoporosis^8^. The F^−^ ions are also used for the separation of radioactive and non-radioactive substances in nuclear industry^9^. Apart from its biological activity, the F^−^ ions are also used as potential catalysts in synthesis and is identified as a strong Lewis’s base^10^. In addition, the F^−^ ions have also various industrial application mainly in steel and aluminum industries^11^. The rate of absorption of the F^−^ ions are fast as compared to excretion^12^. The World Health Organization (WHO) has set the extreme limit of F^−^ ions (1.5 mg L^−1^) for human health^13^. The high intake of the F^−^ ions cause fluorosis which is supplemented with set of diseases such as tooth mottling, metabolic disturbances and neurotoxicity^14^. In molecular and cell biology, a greater concentration of NaF also affects the cell signaling processes and thus causes apoptosis^15^. Besides this, it was also expected that the F^−^ ions with higher concentration cause mitochondrial disorder and promote mitochondrial oxidative stress^16^. Similarly, the CN^−^ ions are widely known as toxic and extremely effective species to human health. Though, the CN^−^ ions are extensively used in industrial developments, such as artificial plastic and gum industry, production of pharmaceutical, gold-silver mining, metallurgy and X-ray film recovery^17,18^. In nature, various food and plants also contain CN^−^ ions^17^. In chemical industry, the CN^−^ salt (1.5 million ton/year) are also involved in synthesis of nitriles, nylon, and acrylic polymer^19^. Even at low concentrations, the CN^−^ ions are extremely toxic^20^. The CN^−^ ions are quickly absorbed through digestive, respiratory and cutaneous paths as a hypertonic anion^21,22^. The increased concentration of CN^−^ ions blocks the electron transport chain and inhibits cellular respiration. The maximum concentration of the CN^−^ ions in drinking water is 1.9 µM as defined by WHO^18^. Therefore, the detection of these ions is important^23^. Recently, some colorimetric are reported for the selective and sensitive detection of F^−^ and CN^−^ ions^24–29^. The colorimetric method (naked-eye detection) is typically based on N–H proton transfer from donor unit to anions. The example of proton donors include urea and pyrrole derivatives^30–34^.

Quinoline and its derivatives have been utilized for designing probes for various ions because of their high chelating ability and unique photophysical properties^35–41^. Herein, we have reported three quinoline derivative-based chemosensors RB-1, RB-2 and RB-3 for the detection of F^−^ and CN^−^ ions. These chemosensors were synthesis in a single step by condensation of quinoline aldehyde with substituted thiosemicarbazides. The synthesized chemosensors were thourghly characterized by NMR (^1^H and ^13^C) and electronic absorption (UV–Vis and FTIR) spectroscopies. The remarkable color changes of the chemosensors with the addition of both fluoride as well as cyanide ions were observed. The Computational calculations were executed for confirmation of experimental findings using FMO, UV–Vis, QTAIM and global reactivity parameters (GRPs) elucidation.

## Experiment

### Methods and material

The chemicals purchased from Sigma Aldrich were used without further purifications. All of the reactions took place in an oven-dried round bottom flask. Thin layer chromatography (TLC) was used to track the reaction progress on a 0.25 mm thick silica plate, and the chromatograms were visualized using staining agents or UV illumination (254 nm). The tetra butyl ammonium salts of fluoride, iodide, chloride, perchlorate, bromide, acetate, bisulfate, thiocyanate and bisulfate were used for the preparation of solutions. The ^1^HNMR spectra were collected on a Bruker spectrometer. The UV–visible measurement were carried on Shimadzu UV-1800 spectrophotometer using 1 cm path length quartz cuvette. The stock solution of various ions were prepared in acetonitrile and DMSO.

### Synthesis

**Scheme 1:**
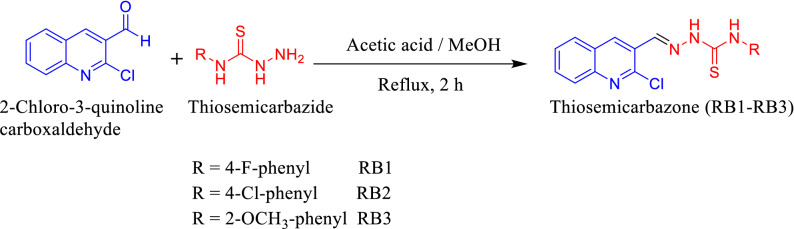
Synthesis of Chemosensors RB1 to RB3.

Thiosemicarbazides were prepared using a formerly described method^42^. In a typical synthesis, the quinoline aldehyde (3.28 mmol) was added in MeOH at 90 °C in the presence of a catalytic (2-3drops) volume of CH_3_COOH (Scheme 1). The appropriate amount of thiosemicarbazide (3.31 mmol) was added to this solution and refluxed for 2–3 h. The precipitates formed were filter and washed with cold methanol after the reaction was validated by TLC. The product RB-1, RB-2 and RB-3 were characterized by NMR (^1^H and ^13^C) and electronic absorption (FTIR) [S.I. 1–9].

#### (E)-2-((2-chloroquinolin-3-yl)methylene)-N-(4-fluorophenyl)hydrazine-1- carbothioamide [RB-1]

Yield, 93%, Melting point, 199–200 °C , ^1^H-NMR (DMSO-*d*^*6*^) δ*ppm*; 7.08 (d, 1H, J = 7.1 Hz, Ar–H), 7.4 (m, 2H, Ar*–*H), 7.6 (dd, 1H, *J* = 7.6, 7.8 Hz, Ar–H), 7.7 (t,1H, Quinoline H), 7.8 (t,1H, Quinoline H), 7.9 (dd, 1H, *J* = 8.0, 7.8 Hz*,* Quinoline H), 8.0 (dd, 1H, *J* = 8.0, 7.1 Hz Quinoline H), 8.65 (s,1H, Quinoline H), 9.37(s, 1H,CH=CN), 12.09 (s, 1H, C=N–N-H) 10.35(s, 1H, N–H-CS);^13^C NMR δ*ppm*; 112.8,113.0, 113.3, 122.2, 126.3, 128.5, 129.0, 130.2, 132.2, 136.8, 138.5, 141.1, 147.5, 149.0, 160.1, 163.3, 176.6. Elemental Analysis calculated for C_17_H_12_ClFN_4_S (358.82); C, 56.91; H, 3.37; N, 15.61; Found; C, 56.98; H, 3.45; N, 15.42.

#### (E)-N-(4-chlorophenyl)-2-((2-chloroquinolin-3-yl)methylene)hydrazine-1- carbothioamide [RB-2]

Yield, 91%, Melting point, 188–190 °C , ^1^H-NMR (DMSO-*d*^*6*^) δ*ppm*; 7.6 (dd 2H *J* = 7 Hz, Ar–H ), 7.7 (dd, 2H, *J* = 7.6 Hz, Ar–H), 7.74 (t,1H, Quinoline, H2), 7.86 (t,1H, Quinoline, H3), 7.9 (dd, 1H, *J* = 8.0 Hz, Quinoline H4), 8.0 (dd, 1H, *J* = 8.0 Hz, Quinoline H1), 8.66 (s,1H, Quinoline H), 9.38(s, 1H, CH=CN), 12.28 (s, 1H, C=N–N-H), 10.35(s, 1H, N–H-CS);^13^C NMR δ*ppm*; 112.8,113.0, 126.4, 127.3, 128.3, 128.6, 129.0, 130.2, 132.2, 136.8, 138.3, 138.4, 147.5,149.0, 176.9. Elemental Analysis calculated for C_17_H_12_Cl_2_N_4_S (375.27); C, 54.41; H, 3.22; N, 14.93; Found; C, 54.37; H, 3.15; N, 15.01.

#### (E)-2-((2-chloroquinolin-3-yl)methylene)-N-(2-methoxyphenyl)hydrazine-1- carbothioamide [RB-3]

Yield of the compound 92%, Melting point, 203–205 °C, ^1^H-NMR (DMSO-*d*^*6*^) δ*ppm*; 3.79 (s 3H Ar-CH3), 7.2 (d 1H *J* = 7.1 Hz*,* Ar–H), 7.2 (m, 2H, Ar–H), 7.72 (t,1H, Quinoline H2), 7.8 (t, 1H, Quinoline, H3), 7.9 (dd, 1H, *J* = 8.0 Hz, Quinoline H4), 8.07(dd, 1H, *J* = 8.0 Hz, Quinoline H1), 8.65 (s, 1H, Quinoline H), 9.41(s, 1H,CH=CN), 12.02 (s, 1H, C=N–N-H), 10.2(s, 1H, N–H-CS);^13^C NMR δ*ppm*; 40.5,40.3,39.9 55.69, 111.6, 112.2, 118.7, 126.4, 127.2, 129.4, 136.8,140.4, 147.5,149.0,159.5,163.11,176.51. Elemental Analysis calculated for C_18_H_15_ClN_4_OS (380.86); C, 58.30; H, 4.08; N, 15.11; Found; C, 58.37; H, 4.15; N, 15.03.

### Computational procedure

The sensing behaviors of RB-1, RB-2 and RB-3 chemosensors towards fluoride ions recognition were investigated theoretically using DFT and TDDFT calculations. Gauss View 5.0^43^ generated input files of RB-1, RB-2 and RB-3 chemosensors were subjected to Gaussian 09 W^44^ program package. To find appropriate functional for performing the entire computational analysis of current draft, geometry optimization analysis was initially carried out at various levels of DFT like B3LYP^45^, CAM-B3LYP^46^, LC-BLYP^47^, M06^48^, M06-2X^49^ and 6-311G(d,p) basis set combinations. The completion of optimization analysis and presence of optimized geometries at lower energy minima is confirmed through frequency analysis using same above-mentioned combinations of functionals. The stability of the optimized geometries was confirmed due to the absence of imaginary frequency. Singlet states in RB-1, RB-2 and RB-3 chemosensors were calculated by performing TDDFT computations in acetonitrile solvent using CPCM model at B3LYP/6-311G(d,p), CAM-B3LYP/6-311G(d,p), LC-BLYP/6-311G(d,p), M06/6-311G(d,p), and M06-2X/6-311G(d,p) functionals. The TDDFT computed absorption maxima (λ_max_) values at M06/6-311G(d,p) level of theory were found in good agreement with experimental λ_max_ values of RB-1, RB-2 and RB-3. Therefore, M06/6-311G(d,p) level is selected for further quantum chemical calculations of RB-1, RB-2 and RB-3. Geometry optimization followed by the frequency analysis of RB-1, RB-2 and RB-3 with F^−^ ions is executed at M06/6-311G(d,p) level of theory. Frontier molecular orbital (FMO), and QTAIM analysis were performed using same selected functional.

## Results and discussion

### UV–Vis spectral studies of chemosensors with fluoride ions

**Figure 1:**
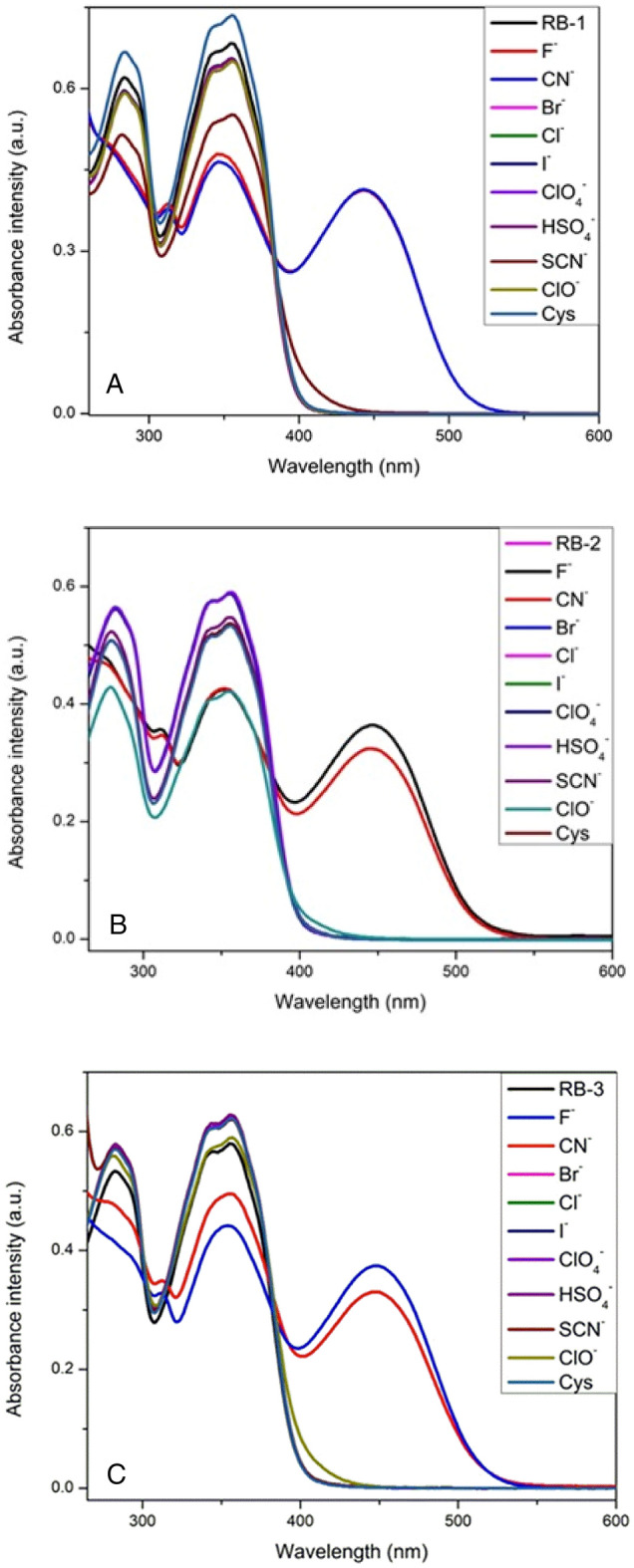
(**A–C**) UV–vis spectra of chemosensors RB-1, RB-2 and RB-3 in CH_3_CN after addition of anions (10 µM).

The behavior of chemosensors (RB-1, RB-2 and RB-3) toward anions recognition such as the F^−^, Br^−^, Cl^−^, I^−^, AcO^−^, CN^−^, HSO_4_^−^, ClO_4_^−^, SCN^−^, ClO^−^, and Cys were studied by observing changes in electronic absorption spectra of chemosensors (10 μM) in acetonitrile (CH_3_CN). The UV–Vis spectra of pure RB-1, RB-2 and RB-3 showed an absorbance band at 350 nm along with a shoulder peak at approximately 285 nm as depicted in Fig. 1A–C. These absorbance bands are reduced and new absorbance band centered around 445, 450 and 450 nm were emerged for RB-1, RB-2 and RB-3 respectively. We also observed vibrant isosbestic point approximately at 383, 382 and 384 nm when F^−^ and CN^−^ ions were added to the solutions of respective chemosensors. However, there were negligible changes with hypochlorite ions and almost no changes were observed with other anions. The reaction between chemosensors and F^−^ ions were completed within 2 s. These chemosensors were used as a naked eyes sensors as the color of solutions changed from colorless to yellow [S.1.10].

### UV–vis titration with fluoride ions

**Figure 2:**
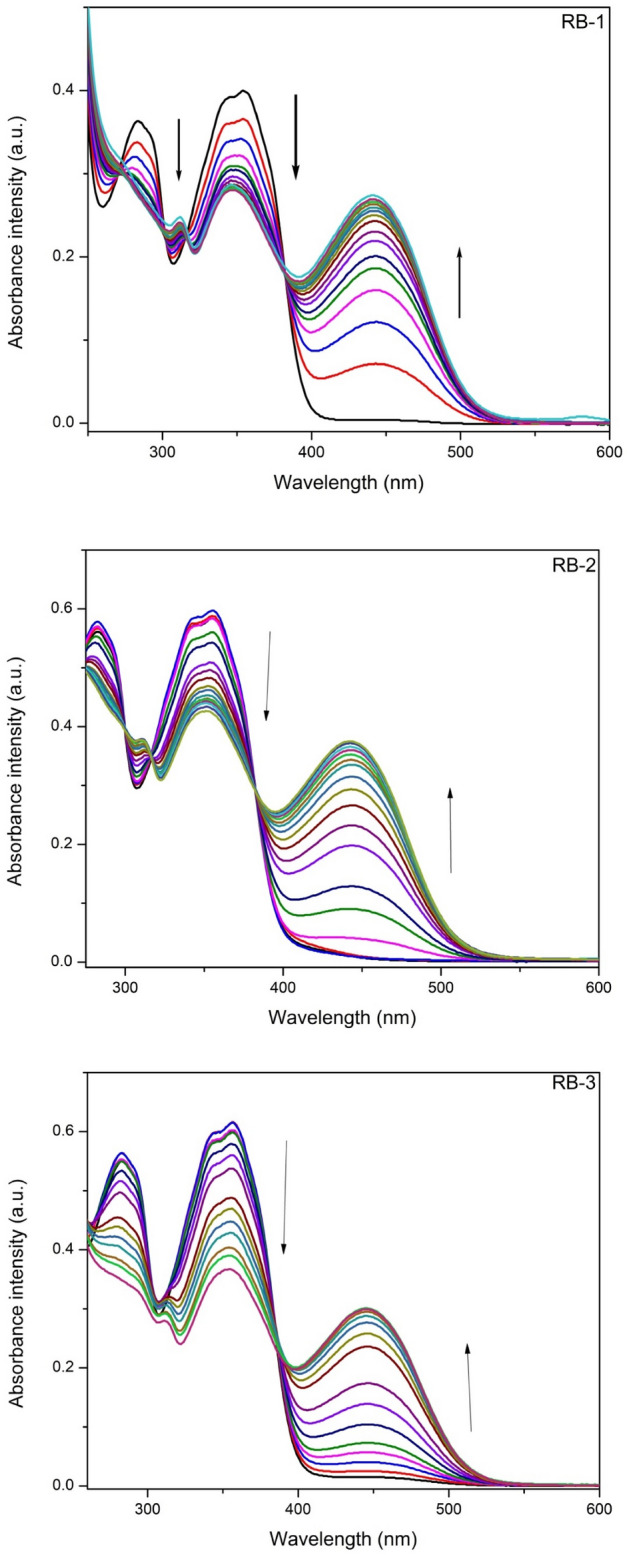
UV–Vis titration spectra of chemosensors RB-1, RB-2 and RB-3 in CH_3_CN after addition of F^−^ ions (12 equiv.).

To confirm the interaction between these chemosensors and F^−^ ions, the UV–Vis titrations of chemosensors were recorded in CH_3_CN by adding different amounts of F^−^ ions. The absorption bands present at 350 nm along with shoulder peak at approximately 285 nm were gradually decreased with the new bands appearing at 445, 450 and 450 nm. These new absorption bands at 445, 450 and 450 were gradually enhanced and reached their saturation points when 12 equiv. of F^−^ ions were added (Fig. 2). By using the Benesi-Hildebrand equation (Eq. 1) the binding constant [BH plot S.I. 11–13] of all chemosensors with F^−^ ions were calculated. The limits of detection (LOD) calculated by using equation (LOD = 3σ/slope) [linear plot S.1. 14–16] are summarized in Table 1.

**Table 1:** Comparison of Limits of detection and binding constants of receptors RB-1 to RB-3 with other reported sensors.

References	Structure	Binding constant (M^−1^)	Detection limit
^55^		4.48 × 10^3^	9.08 × 10^−5^
^56^		4.92 × 10^4^	23.33 × 10^–6^
^57^		–	1 × 10^−4^
^58^		1.93 × 10^4^	20.5 × 10^−6^
^59^		1.39 × 10^4^	22.54 × 10^−6^
This work		1.53 × 10^4^ 7.30 × 10^4^ 1.11 × 10^4^	9.60 × 10^−6^ 12.70 × 10^−6^ 12.05 × 10^−6^

Benesi—Hildebrand equation

1 $$\frac{b}{\Delta A} = \frac{1}{StKa\Delta \upvarepsilon} \times \frac{1}{\left[ L \right]} + \frac{1}{St\Delta \upvarepsilon}$$

Δ***A*** = ***A***_substrate + anion_ − ***A***_substrate,_ St = Total concentration of substrate; K_a_ = Binding constant or Association constant; Δε = ε_substrate + anion_ − ε_substrate_ − ε_anion_.

### UV–Vis titration with cyanide ions

**Figure 3:**
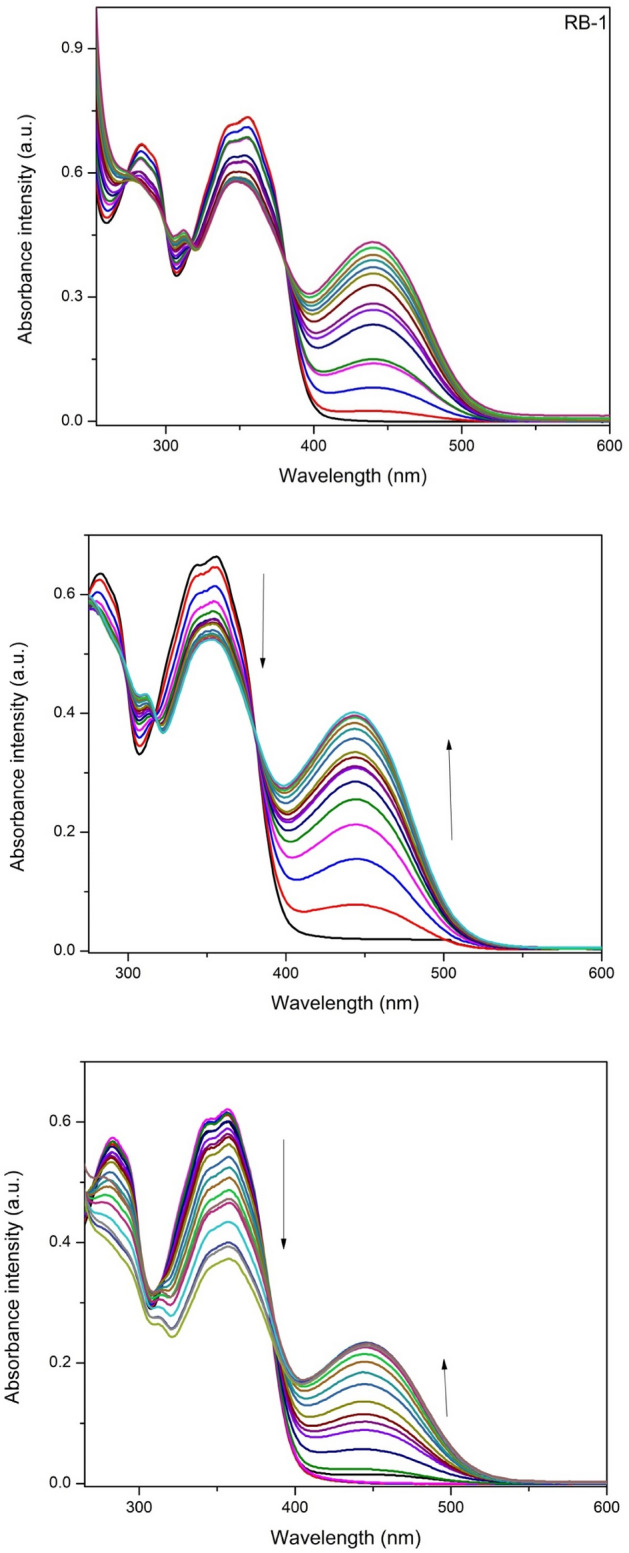
UV–Vis titration spectra of chemosensors RB-1, RB-2 and RB-3 in CH_3_CN after addition of CN ions (12 equiv.).

Similar to F^−^ ions, titration experiments were plaid to check the interaction between chemosensors and CN ions. When solution of all three chemosensors were treated with different amounts of cyanide ions the band centered at 350 nm was reduced gradually with the enhancement in absorbance band at 445, 450 and 450 nm (Fig. 3). By using Benesi-Hildebrand equation (Eq. 1) the binding constant [BH plot S.I. 17–19] of chemosensors with CN^−^ ions was evaluated based on UV–Vis titration while limits of detection (LOD) were calculated by using equation (LOD = 3σ/slope) [linear plot S.1. 20–22] and given below in Table 1.

### Job’s plot

To confirm the binding stoichiometry of chemosensors RB-1, RB-2 and RB-3 we did Job’s plot for all chemosensors with F^−^ and CN^−^ ions and established that the binding ratio is 1:2 [F^−^ : RB-1–3] in all cases [S.I. 23–28]. The results of all performed Job’s plot showed that chemosensors could not distinguish between F^−^ or CN^−^ ions and thus detect both of them. This result indicates that both anions can form adducts with N–H of chemosensors.

### Real-life applications

**Figure 4:**
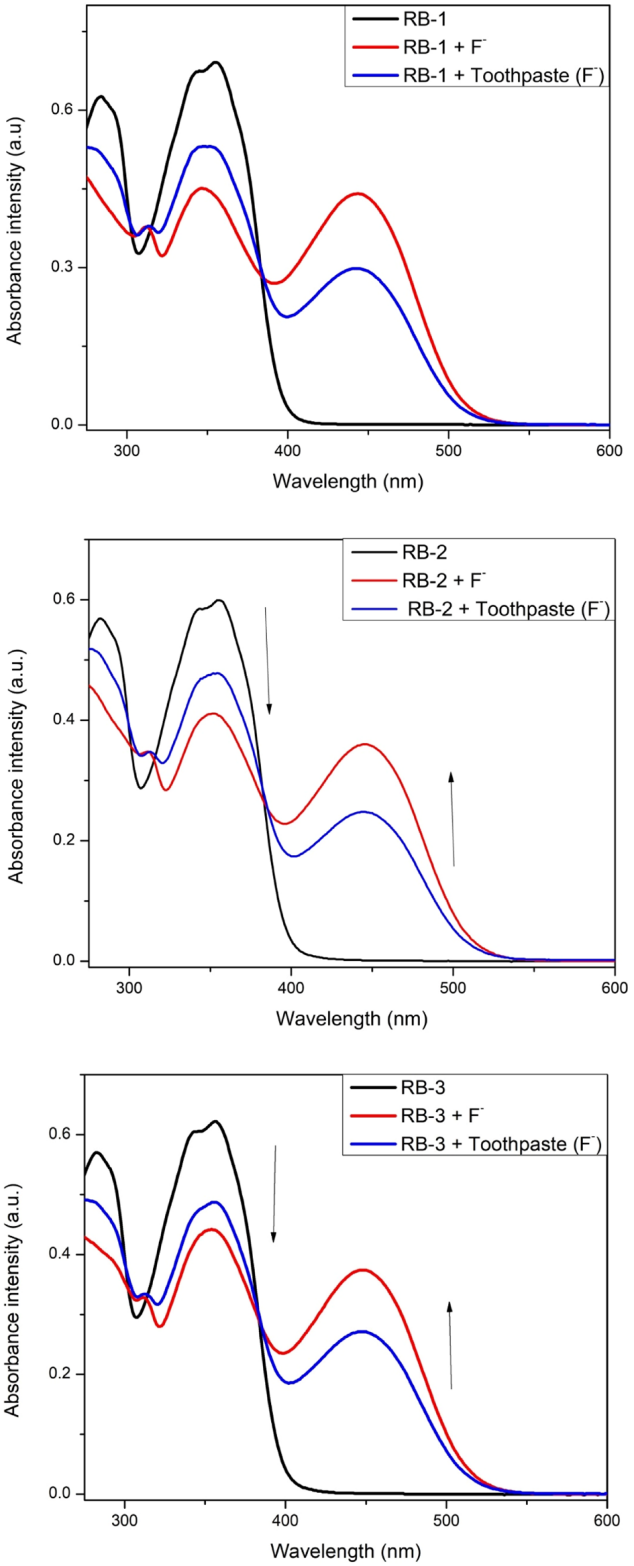
UV–Vis spectra of chemosensors RB-1, RB-2 and RB-3 in CH_3_CN after addition of toothpaste sample (F^−^ ions) (50 µM).

To check the response of these chemosensors to F^−^ ions in real sample, we used commercially available tooth -paste as a source of F^−^ ions. The toothpaste sample for analysis was prepared by dissolving 50 mg of commercially available tooth-paste (Sensodyne) in 3 mL CH_3_CN with sonication followed by centrifugation and filtration. When the filtrate of toothpaste sample (50 µM) was added to chemosensors solutions (10 µM) the bands at 385 were reduced with the emergence of new bands at 445, 450 and 450 nm for RB-1, RB-2 and RB-3 respectively (Fig. 4). The color of solutions was also changed from colorless to yellowish. These observation suggest that our chemosensors can be applied to detect the traces of F^−^ ions in real samples. To further explore the everyday applications, we prepared test strips by soaking Whatman filter paper in acetonitrile solution (1.0 × 10^−3^ M) containing these chemosensors followed by drying in air. When these test strips are treated with F^−^ ions solution, they showed a significant color change [S.I. 29].

### DNA binding study

**Figure 5:**
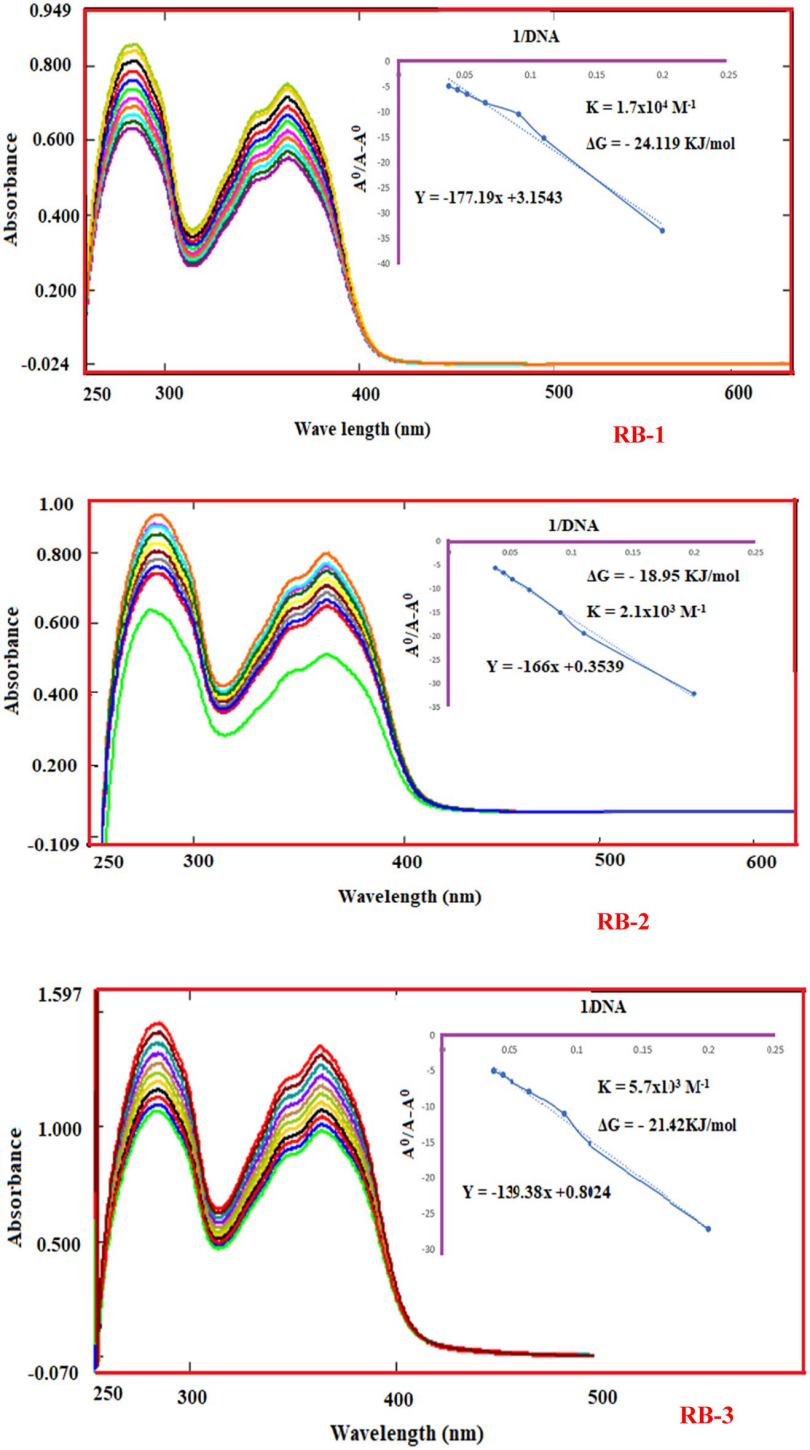
Absorption spectra of RB-1, RB-2 and RB-3 with SS-DNA and graph between A^0^/A-A^0^ vs 1/DNA showing binding constant by the interaction between SS-DNA and the corresponding compounds.

A solution of Salmon sperm’s DNA (SS-DNA) was made in deionized water by overnight stirring and was kept in a buffer to maintain the pH at 4 °C. Absorbance ratio of SS-DNA is set 1.8 by taking absorbance at 260 and 280 nm to keep DNA free from protein^50^. The solution of DNA was titrated against a fixed concentration of chemosensors (1 mM) and absorption measurement were taken at regular intervals as showen in (Fig. 5). The absorption data showed significant hypochromic shifts by the addition of SS-DNA that we speculate is caused by intercalative mode of binding though. π-stacking between SS-DNA and title compounds^51^. For RB-1, RB-2 and RB-3 the binding constant (K) were found to be 1.7 × 10^4^ M^-1^, 2.1 × 10^3^ M^-1^ and 5.7 × 10^3^ M^-1^ respectively. The Gibbs’s free energy (ΔG) values were also calculated by using the equation as:

$$\Delta {\text{G}} = - {\text{RT}}\ln {\text{K}}$$

where R represents the ideal gas constant (8.314 J/K mol) and T indicates absolute temperature (298 K). The Gibb’s free energy (ΔG) values for RB-1, RB-2 and RB-3 were found to be − 24.119, − 18.95 and − 21.42 kJ/mol respectively. The sign of free energy indicates that the interaction between the forementioned compounds with SS-DNA is a spontaneous process.

### Reversibility of reaction

**Figure 6:**
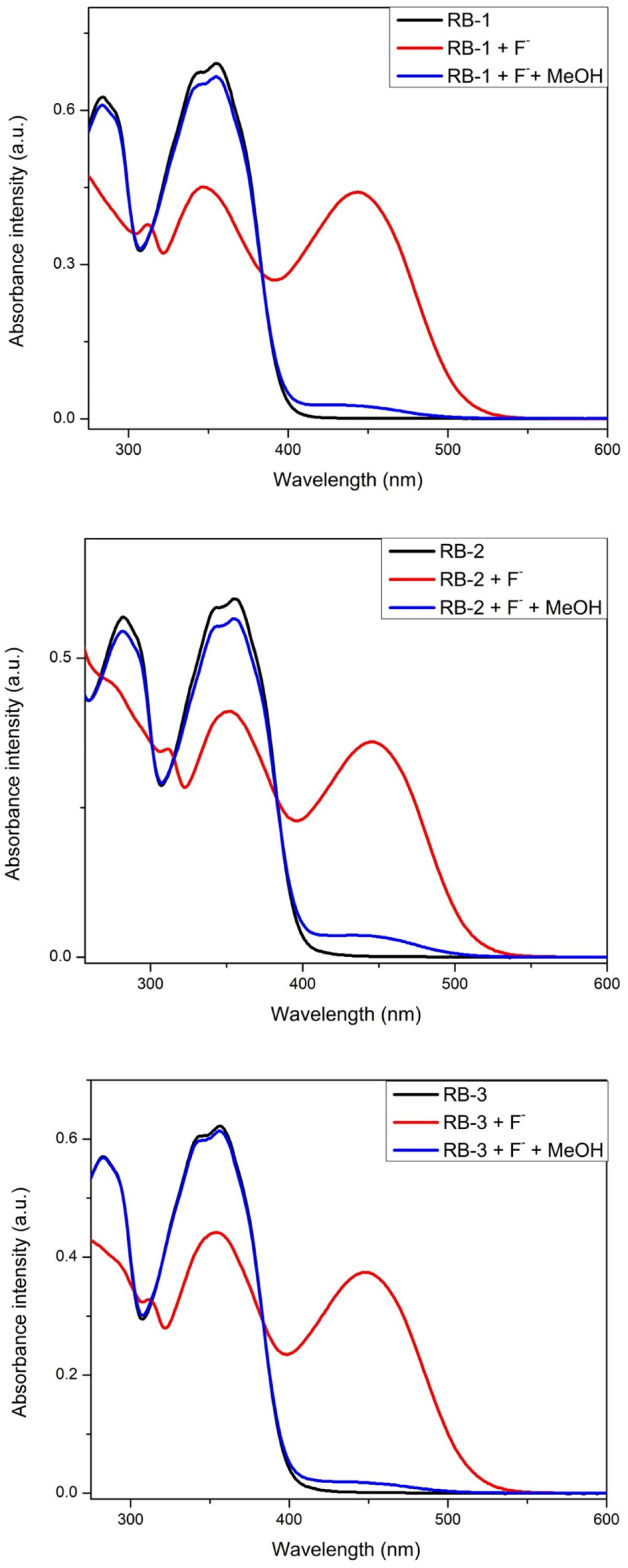
Electronic absorption spectra of chemosensors (RB-1, RB-2 and RB-3) in presence of F^−^ ions and MeOH (0.1 ml).

Methanol is a good proton donor as compared to N–H group of chemosensors (RB-1, RB-2 and RB-3) thus reversible reaction with the addition of methanol was expected as reported somewhere else^52,53^. When 0.1 ml of methanol was added to solutions of chemosensors and fluoride ions, yellow color of the solution was disappeard which indicates the re-protonation of chemosensors (Fig. 6). And the electronic absorption spectra were also reversed to original bands of chemosensors at 350 nm and 285 nm. This observation suggests thst methanol interaction between chemosensor and F^−^ ions can be reversed by the addition of a stronger proton donor.

### Sensing mechanism

**Figure 7:**
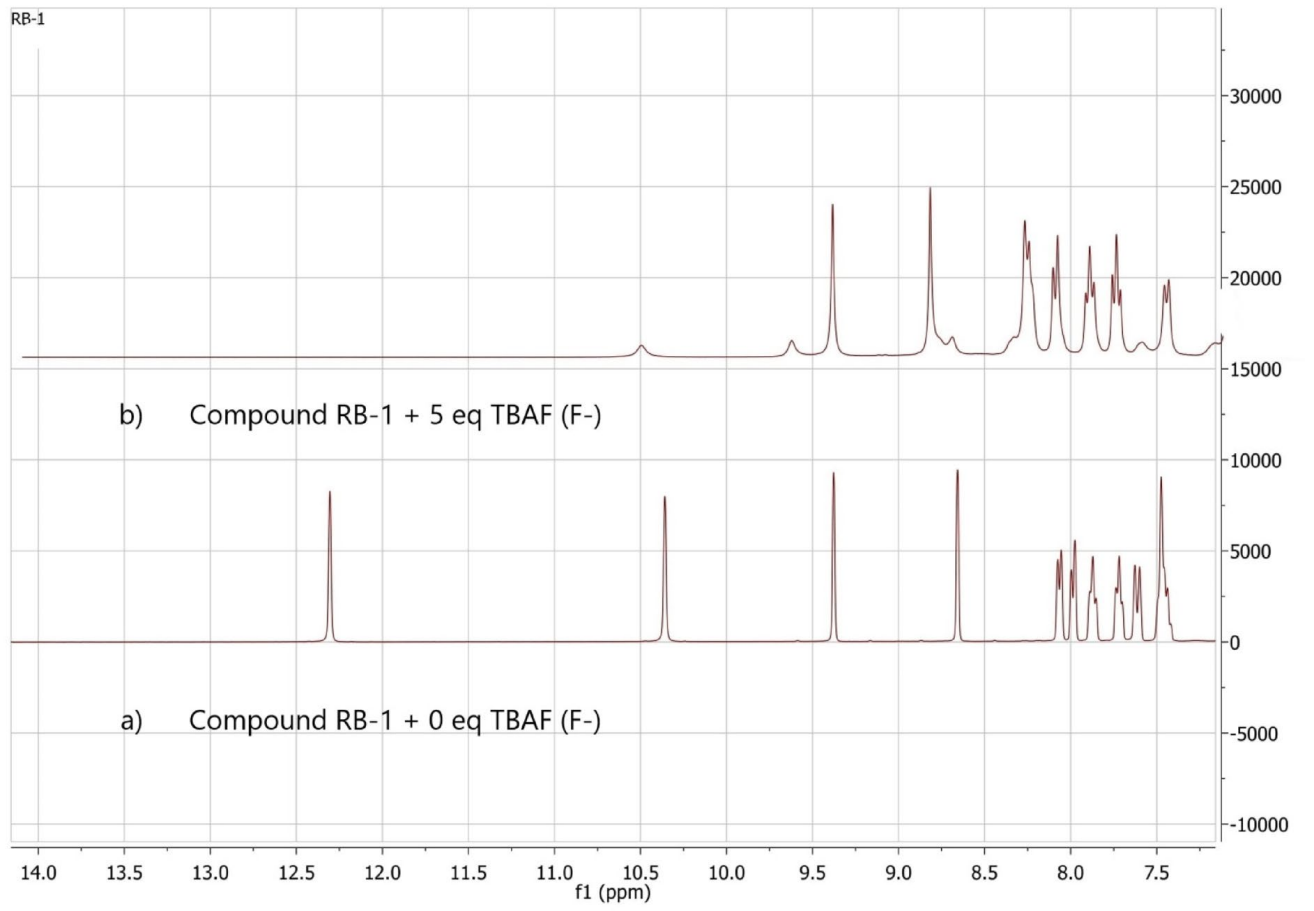
Study of ^1^H-NMR spectra of (RB-1) on addition of Fˉ ions a) no Fˉ ions addition b) 5 equiv. addition in DMSO-d_6_ as solvent.

**Scheme 2:**
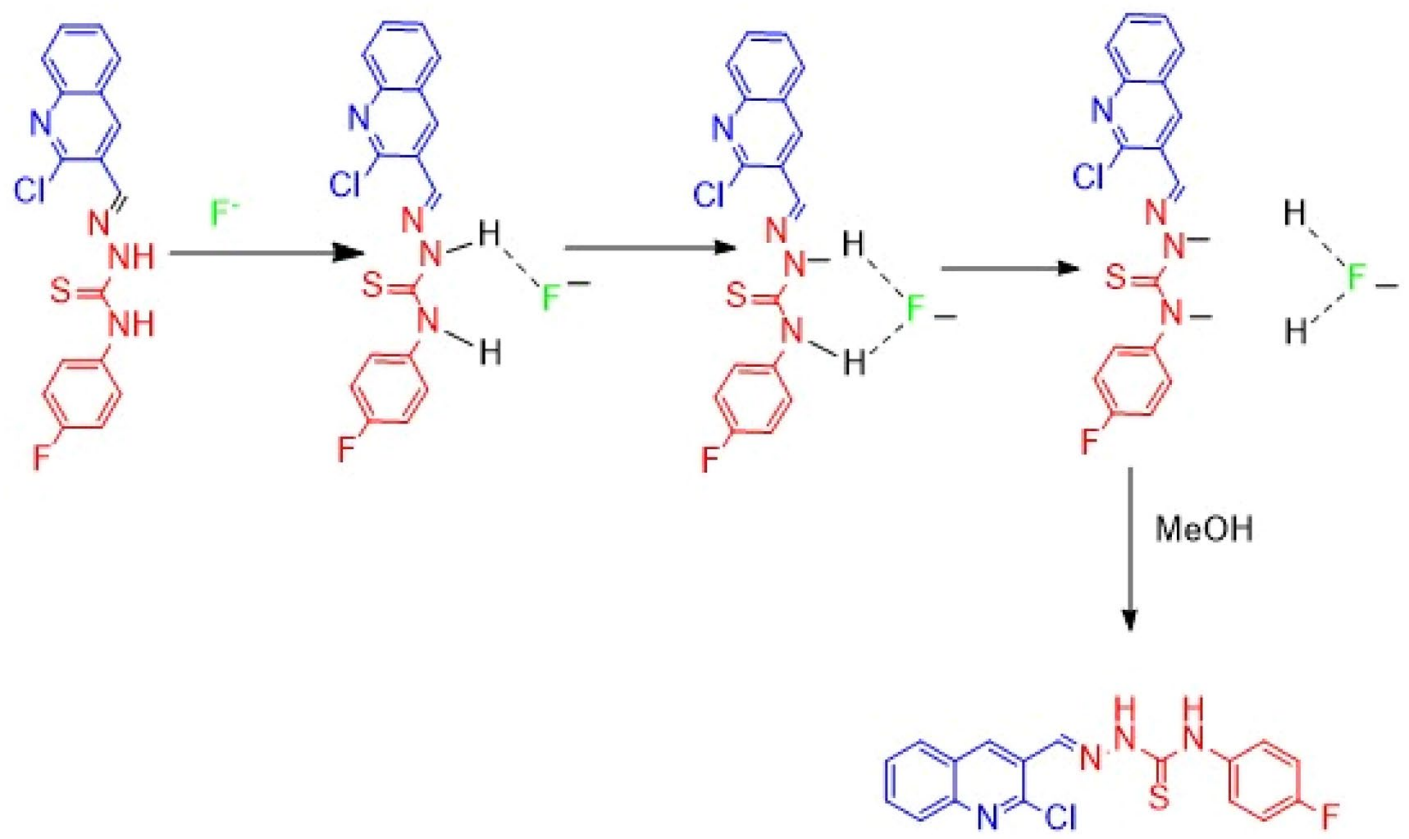
Proposed sensing mechanisms of chemosensor.

^1^HNMR and IR spectroscopy [S.I. 29] were engaged to confirm the proof of the proposed mechanism of chemosensor (RB-1) interaction with F^−^ ions. Tetra-n-butylammonium fluoride (TBAF) was used as source of Fˉ ions and it was slowely added to chemosensor RB-1 solution in DMSO-d_6_. The ^1^H-NMR spectrum of (RB-1) endorses presence of N*H* protons as sharp singlets at *δ* 12.30 and *δ*10.35 ppm. The vanishing of NH protons when F^−^ ions were added indicates that F^−^ ions abstract NH proton (Fig. 7). The proposed sensing mechanism^54^ is illustrated in (Scheme 2).

## Theoretical study

**Figure 8:**
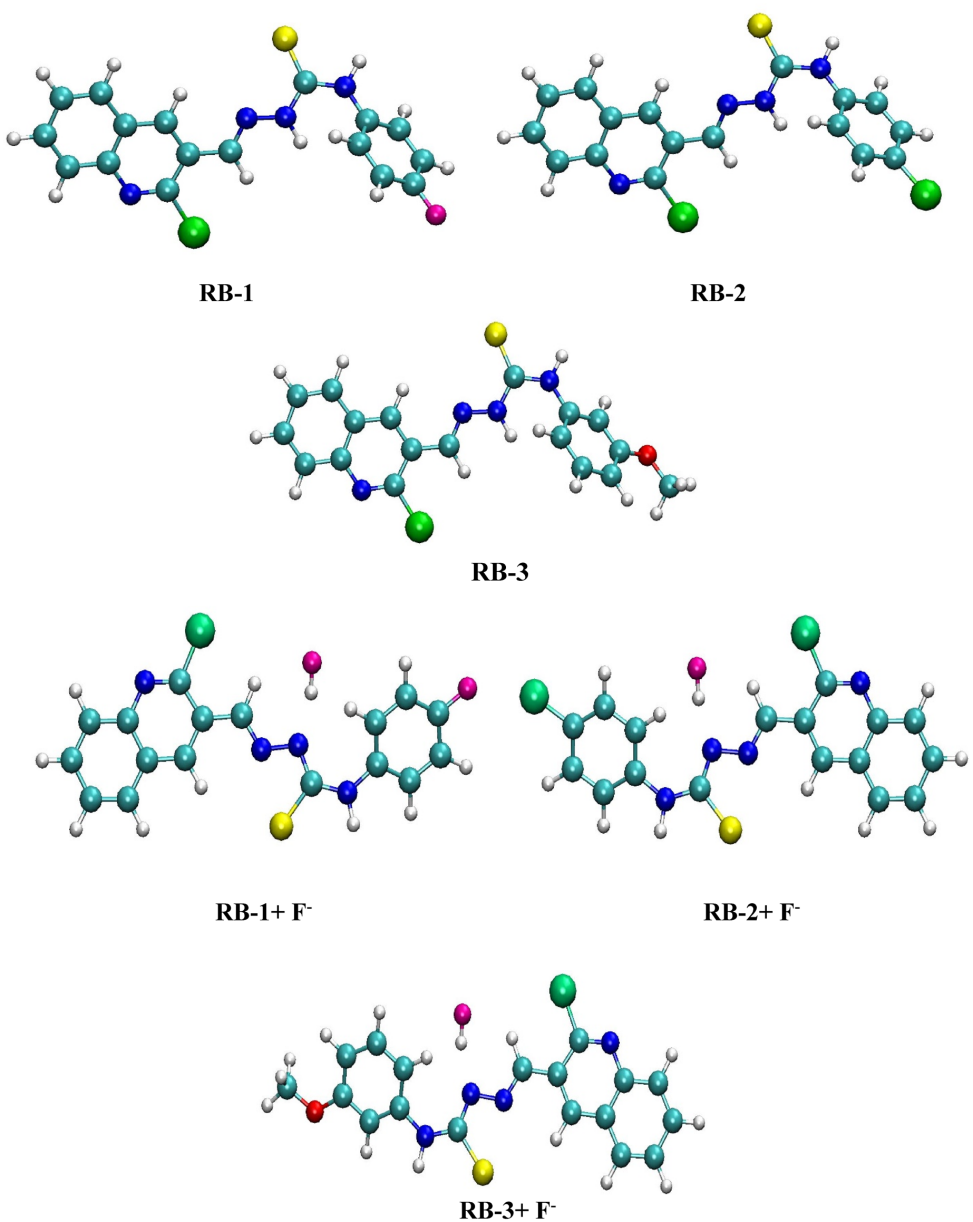
Optimized geometries of RB-1, RB-2 and RB-3 chemosensors without and with F^−^ ions calculated at M06/6-311G(d,p) level of theory.

The optimized geometries of RB-1, RB-2 and RB-3 chemosensors in the absence and presence of F^−^ ions are presented in (Fig. 8).

The bond distance for N(14)-H(31) is significantly enhanced from 1.021 Å to 1.732 Å in both RB-1 + F^−^ and RB-2 + F^−^ complexes due to formation of H(31)-F(37) bonds that have bond lengths of 0.945 Å. The N(13)-N(14) and N(14)-H(30) bond lengths in RB-3 are enlarged from 1.339 and 1.021 Å to 1.371 Å and 1.734 Å..

The calculated energy values of RB-1, RB-1 + F^−^, RB-2, RB-2 + F^−^, RB-3, RB-3 + F^−^ chemosensors and complexes are found to be − 1831.794083 E_h_, − 1931.328578 E_h_, − 2192.149256 E_h_, − 2291.687604 E_h_, − 1847.049344 E_h_, − 1946.585230 E_h_ respectively. These values point out that complexes of chemosensors with F^−^ ions are relatively more stable compared to free chemosensors. The high stability of chemosensors toward F^−^ ions are also supported by experimental spectral analysis of RB-1 + F^−^ ions, RB-2 + F^−^ ions and RB-3 + F^−^ ions_._

### Frontier molecular orbital (FMO) analysis

FMO analyses were performed on both the free and complexes of chemosensors to have useful insight into density distribution and the response of chemosensors toward F^−^ ions detection.

**Figure 9:**
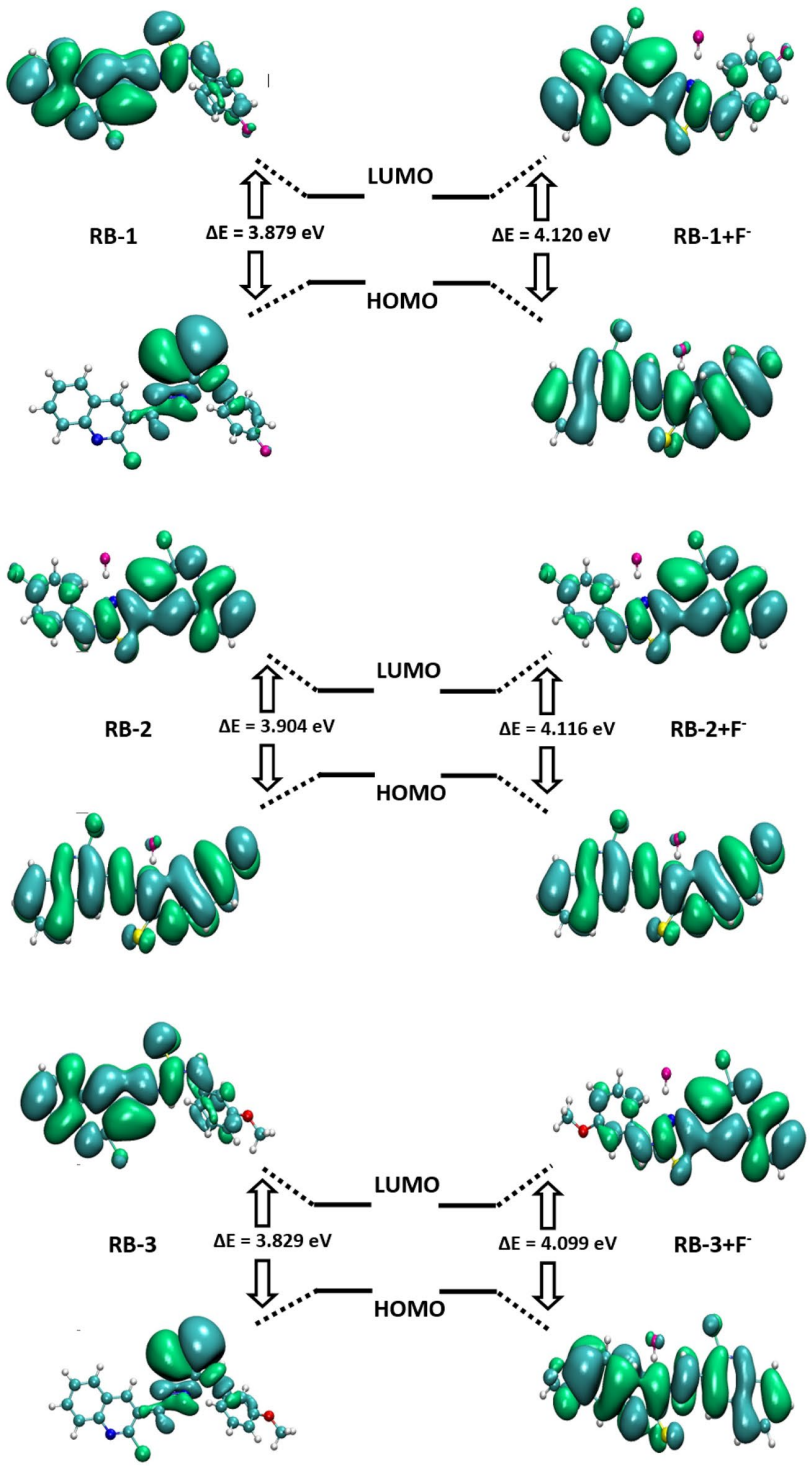
FMOs of RB-1, RB-1 + F^−^, RB-2, RB-2 + F^−^, RB-3, RB-3 + F^−^.

Among free chemosensors, the lowest energy gap value (3.829 eV) is measured in RB-3, while highest energy gap is marked as 3.904 eV in RB-2. The backbone of RB-1, RB-2, RB-3 contains Cl, S, and N atoms at the same position. The only difference is the presence of F, Cl and OCH_3_ units on the terminal benzene ring respectively. These units cause the difference in the energy gap values of free chemosensors. The lowest E_g_ value in RB-3 might be attributed to the presence of moderately activating OCH_3_ groups which form a push–pull configuration with weakly deactivating Cl in RB-3. The highest energy gap in RB-2 is occurred owing to the presence of same weakly deactivating Cl atoms in backbone as well as on terminal benzene ring (Fig. 9).

The outcomes summarized in Table 2 confirm that the addition of F^−^ ions in free chemosensors results in the stabilization of both LUMOs and HOMOs ofchemosensors. The HOMOs and LUMOs of RB-1 + F^−^ ions, RB-2 + F^−^ ions, RB-3 + F^−^ ions are found with increased and stabilized values of − 6.397, − 6.454, − 6.299 eV compared to free chemosensors with − 2.277, − 2.338, − 2.200 eV. The stabilization of HOMOs and LUMOs in complex chemosensors indicates the selective sensing of F^−^ ions with RB-1, RB-2, and RB-3.

**Table 2:** The energy values of HOMO, LUMO of RB1-RB3 chemosensors without and with F^−^ ions.

Molecules	E_HOMO_	E_LUMO_	E_g_
RB-1	− 6.169	− 2.290	3.879
RB-2	− 6.234	− 2.330	3.904
RB-3	− 6.042	− 2.213	3.829
RB-1 + F^−^	− 6.397	− 2.277	4.120
RB-2 + F^−^	− 6.454	− 2.338	4.116
RB-3 + F^−^	− 6.299	− 2.200	4.099

E_g_ = E_LUMO_ − E_HOMO_.

### Global reactivity parameters (GRPs)

Global reactivity parameters of free and complex chemosensors RB-1, RB-1 + F^−^ ions, RB-2, RB-2 + F^−^ ions, RB-3, RB-3 + F^−^ ions are estimated using energy values of FMOs and results are collected in Table 3.

**Table 3:** Global reactivity parameters for RB-1, RB-1 + F^−^ ions, RB-2, RB-2 + F^−^ ions, RB-3, RB-3 + F^−^ ions chemosensors for free and complex.

	IP	EA	X	*μ*	*η*	S	*ω*
RB-1	6.169	2.290	4.229	− 4.229	1.939	0.2577	4.611
RB-2	6.234	2.330	4.282	− 4.282	1.952	0.2561	4.696
RB-3	6.042	2.213	4.127	− 4.127	1.914	0.2611	4.449
RB-1 + F^−^ ions	6.397	2.277	4.337	− 4.337	2.060	0.2427	4.565
RB-2 + F^−^ ions	6.454	2.338	4.396	− 4.396	2.058	0.2429	4.695
RB-3 + F^−^ ions	6.299	2.200	4.249	− 4.249	2.049	0.2439	4.405

*IP*, ionization potential; *EA*, electron affinity; X, electronegativity; *μ*, chemical potential; *η*, hardness; *S*, softness; ω, electrophilicity.

Global softness and hardness are crucial GRPs that are used to describe the chemical nature of the molecules under investigation. It can be seen from Table 3 results that softness values in free chemosensors are larger compared to complexed chemosensors. This implies that the stability of chemosensors is increased in the presence of F^−^ ions. Similar results are marked in case of hardness values which are found in the range of 1.914–1.952 in the absence of ions and 2.049–2.060 in the presence of ions. Global hardness results also favor the stance that chemosensors RB-1, RB-2, RB-3 upon addition of fluoride ions convert into kinetically stable, hard and less reactive chemosensors. Larger IP values of complexed chemosensors RB-1 + F^−^ ions, RB-2 + F^−^ ions, RB-3 + F^−^ ions as compared to free RB-1, RB-2, RB-3 chemosensors tell the same story of more stability, less reactivity and their hard nature. From electronegativity and chemical potential results, it can be inferred that increased values of *X* and decreased values of *μ* in RB-1 + F^−^ ions, RB-2 + F^−^ ions, RB-3 + F^−^ ions than RB-1, RB-2, RB-3 is due to the formation of H–F bonds. Overall, the presence of stability, hard nature, and less reactivity of RB-1 + F^−^ ions, RB-2 + F^−^ ions, RB-3 + F^−^ ions point out that investigated chemosensors RB-1, RB-2, RB-3 are suitable candidates for sensing the fluoride ions.

### UV–Vis spectral analysis

TDDFT computations were carried out to interpret the changes in free chemosensors when they are complexed with F^−^ ions. In the absence of ions, the absorption bands at 355 and 287 nm in RB-1 displayed excellent agreement with experimentally observed bands at 350 and 285 nm (Table 4). The TDDFT computed absorption bands mainly correspond to HOMO-1 → LUMO and HOMO-1 → LUMO + 1 respectively. Similarly, DFT computed λ_max_ values of 355 and 289 nm were calculated for RB-2 in agreement with experimental values. Appropriate agreement between experimental and DFT computed λ_max_ values is also seen for RB-3. The oscillator strength values of free chemosensors are found higher than complexed chemosensors. The intense band in RB-1 + F^−^ ions at 542 nm that arises with a moderate value of oscillator strength (0.0194) is red-shifted as compared to RB-1. Similar intensification of λ_max_ values of RB-2 from 355 to 541 nm is measured upon addition of F^−^ ions in RB-2. The red-shifted λ_max_ values in RB-2 + F^−^ ions compared to RB-2 is found relatively with lower transition energy and oscillator strength. HOMO-5 → LUMO transitions in RB-3 + F^−^ ions red shifted the absorption band to 577 nm with 2.14 Ex and 0.0333 ƒ_os_ values. Thus, red-shifting of absorption maximum and changes in spectral properties due to formalization of complexes are also well reflected in experimental calculated spectral analyses of free and complex chemosensors.

**Table 4:** UV–Vis spectral analysis of RB-1, RB-1 + F^−^ ions, RB-2, RB-2 + F^−^ ions, RB-3, RB-3 + F^−^ ions chemosensors at TDDFT/M06/6-311G(d,p) functional.

	λ_max_ (nm) DFT	E_x_(eV)	ƒ_os_	Major electron transportation (%)
RB-1	355.08	3.49	0.5742	H-1 → L (95%)
	287.88	4.30	0.4099	H-4 → L (12%), H-3 → L (16%), H-1 → L + 1 (57%)
RB-2	355.22	3.49	0.6234	H-1 → L (95%)
	289.06	4.28	0.3753	H-4 → L (11%), H-3 → L (14%), H-1 → L + 1 (58%)
RB-3	357.03	3.47	0.5894	H-2 → L (11%), H-1 → L (84%)
RB-1 + F^−^ ions	542.67	2.28	0.0194	H-5 → L(20%), H-4 → L (19%), H-2 → L (27%)
RB-2 + F^−^ ions	541.43	2.28	0.0215	H-5 → L (21%), H-4 → L(19%), H-2 → L) (20%)
RB-3 + F^−^ ions	577.79	2.14	0.0333	H-5 → L(62%), H-4 → L(11%)

Exp., Experimental; H, HOMO; L, LUMO.

### QT-AIM analysis

**Figure 10:**
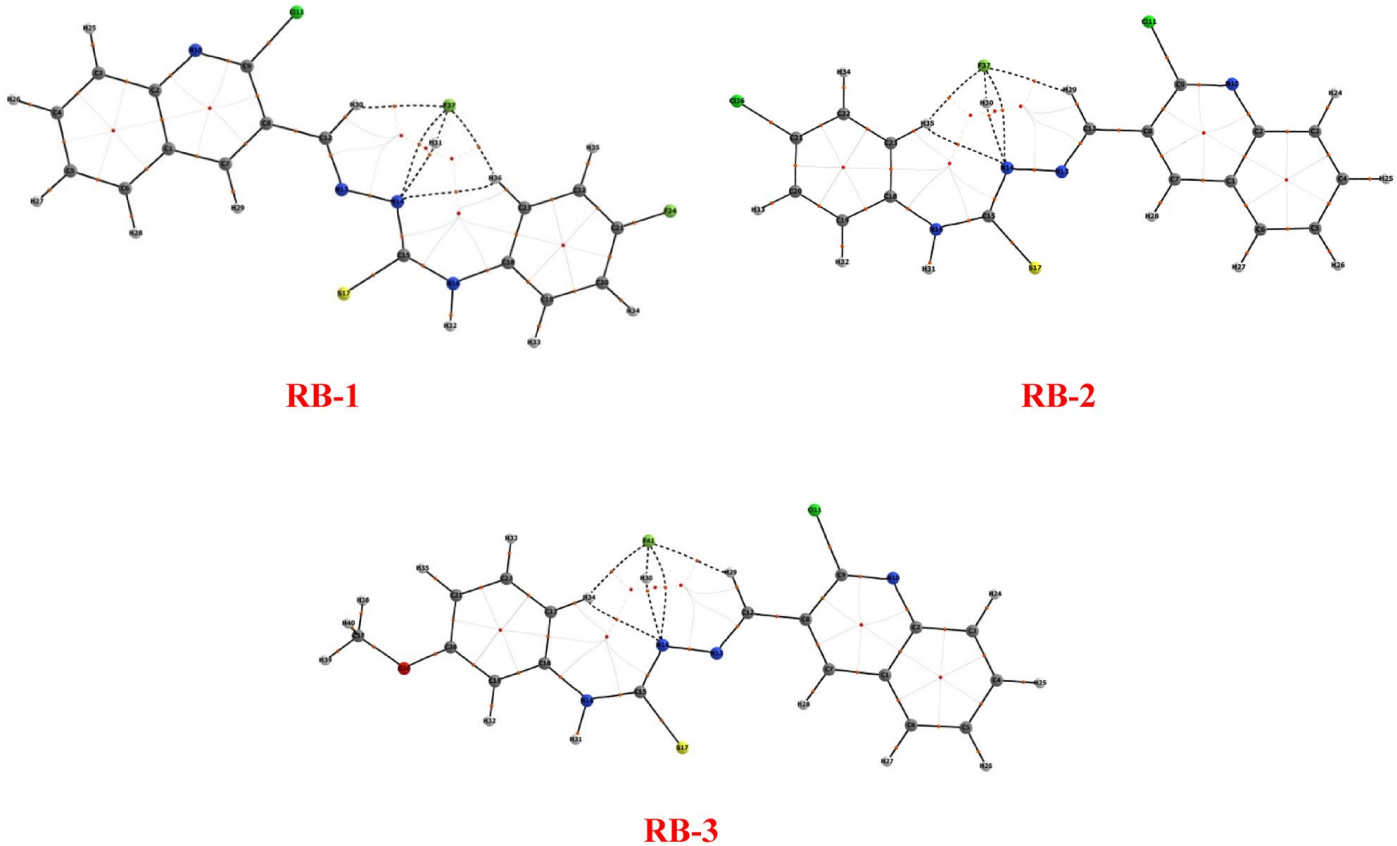
Schematic structure of AIM analyses for RB-1, RB-2 and RB-3 compounds respectively.

QT-AIM study was achieved to assess the intra- and intermolecular non-covalent interactions like hydrogen bonds (HBs) via Theory of Atoms in Molecules^60–62^ for the entitled compounds such as RB-1, RB-3 and RB-1, respectively (Fig. 10). Non-covalent intermolecular interactions gained a lot of attention due to their performance in the maintenance of molecular arrangement. They could manage the intermolecular aggregation processes in terms of the polarity and nature of the involved species^63^.

The non-covalent interactions (NCI) phenomenon is accomplished by calculating real-space regions where non-covalent interactions are necessary and depend altogether on ρ and its gradient^64^. Strong hydrogen bonding (HBs) such as, (N–H⋯O, N–H⋯N, O–H⋯N and O–H⋯O etc.) are basic in crystal engineering^65,66^. The AIM analysis showed that the molecules having inter- and intra-molecular interactions are stable and these interactions can be observed by the dashed bond path (BPs) between the atoms (Fig. 10).

For entitled compounds, two different sets of HBs existed; one intra-molecular and the other with solvent interaction. The intra-molecular HB exhibition was found between nitrogen of hydrazine moiety and the hydrogen of the benzene ring, as their $\rho$ values at BCPs were found to be + 0.011203 e/a^3^ (N14-H36), and + 0.011622 e/a^3^ (N14-H35) and + 0.011540 e/a^3^ (N14-H34) for RB-1, RB-2 and RB-3 respectively. The solvent-based HBs were much less strong in comparison to the intra-molecular HBs.

Furthermore, for the RB-1 compound, AIM approach confirmed the presence of the intermolecular (C–H…F) hydrogen bonds, as the $\rho$ values at BCPs H30-F37, H36-F37 and H31-F37 were + 0.011702, + 0.009453 and + 0.020995 e/a3 respectively. Similarly, the presence of intermolecular (C–H…F) hydrogen-bonds, the $\rho$ values at BCPs H29-F37, H35-F37 and H30-F37 were + 0.011652, + 0.009795 and + 0.021013 e/a3 for RB-2 and H29-F41, H34-F41 and H30-F41 were + 0.012629, + 0.008256 and + 0.020777e/a3 for RB-3 respectively. QTAIM analysis also proposed the existance of C–N…F and N–H…F interactions in RB-1 as the *ρ* values at BCPs N14-F37 and N14-H31 were + 0.007210 and + 0.007573 e/a3 respectively (Table S.1. 30). Similarly, the *ρ* values at BCPs N14-F37 and N14-H30 were + 0.007226 and + 0.007589 e/a3 for RB-2 and N14-F41 and N14-H30 were + 0.007310 and + 0.007468 e/a3 for RB-3 respectively (Table S.I. 31 & 32).

These interactions clearly showed that this is the strongest NCI present in RB-1, RB-2 and RB-3 (Fig. 10).

## Conclusion

In summary, a series of novel quinoline fluorophore based chemosensors (RB-1, RB-2 and RB-3) for detection of F^−^ and CN^−^ ions were synthesized. When these chemosensors were treated with F^−^ and CN^−^ ions a new absorbance band appeared which can be reinstated to original one by treating with MeOH. Chemosensors presented a very fast comeback to F^−^ ions (2 s) with change in color from colorless to yellow. The detection limit for F^−^ and CN^−^ ions was calculated as 2.46, 2.48 and 2.49 (10^–7^) and 2.03, 2.47 and 2.48 (10^–7^) respectively for RB-1, RB-2 and RB-3. The Binding constant for F^−^ and CN^−^ ions was calculated as 1.53, 7.30 and 1.11 (10^4^ M^−1^) and 1.56, 3.50 and 3.55 (10^4^ M^-1^) respectively for RB-1, RB-2 and RB-3. Chemosensors were also employed to detect the traces of F^−^ ions in toothpaste (Sensodyne). DFT and TDDFT based FMOs results confirmed the selective sensing of F^−^ with RB-1, RB-2, RB-3 as HOMOs and LUMOs of RB-1 + F^−^ ions, RB-2 + F^−^ ions, RB-3 + F^−^ ions are found with increased and stabilized values compared to the free chemosensors. Large hardness values (2.049–2.060) upon sensing the F^−^ as compared to free chemosensors values (1.914–1.952) indicate the less reactive, hard and kinetically stable nature of complex sensors. The red-shifting of absorption maximum values upon sensing the F^−^ ions in RB-1 + F^−^ ions, RB-2 + F^−^ ions, RB-3 + F^−^ ions and changes in spectral properties due to formalization of complex are measured which were also well reflected in experimental calculated spectral analyses of free and complex chemosensors. QTAIM study indicate that H⋅⋅⋅F bond in RB-1 + F^−^ ions, RB-2 + F^−^ ions, RB-3 + F^−^ ions are strong enough which support the sensing of F^−^ via N–H⋅⋅⋅F interactions. We believe that these chemosensors have great potentia for the quantitative analysis in real samples.

## Supplementary Information

Supplementary Information.
