# Supplementary material for: Quinoline based thiosemicarbazones as colorimetric chemosensors for fluoride and cyanide ions and DFT studies
Source: Sci Rep. 2022 Mar 23;12:4927. doi: 10.1038/s41598-022-08860-3 (PMC8943172; doi:10.1038/s41598-022-08860-3)
Supplement: Supplementary file 1 — Supplementary Information. [file 41598_2022_8860_MOESM1_ESM.docx]

Supplementary Information for

**Quinoline Based Thiosemicarbazones as Colorimetric Chemosensors for Fluoride and Cyanide ions and DFT Studies**

Rabia Basri^a^, Nadeem Ahmed^a^, Muhammad Khalid^b^*, Muhammad Usman Khan^c^, Muhammad Abdullah^d^, Asad Syed^e^, Abdallah M. Elgorban^e^, Salim S.Al-Rejaie^f^, Ataualpa Albert Carmo Braga^g^ and Zahid Shafiq^a*^

*^a^ Institute of Chemical Sciences, Bahauddin Zakariya University, 60800 Multan, Pakistan*

*^b^Department of Chemistry, Khwaja Fareed University of Engineering & Information Technology, Rahim Yar Khan, 64200, Pakistan*

*^c^ Department of Chemistry, University of Okara, Okara-56300, Pakistan*

*^d^Department of Chemistry, Colorado State University, Fort Collins, CO 80523, USA*

*^e^Department of Botany and Microbiology, College of Science, King Saud University, P.O. 2455, Riyadh, 11451, Saudi Arabia*

*^f^Department of Pharmacology and Toxicology, College of Pharmacy, King Saud University, P.O.Box 55760, Riyadh 11451 Saudi Arabia*

*^g^Departamento de Química Fundamental, Instituto de Química, Universidade de São Paulo, Av. Prof. Lineu Prestes, 748, São Paulo, 05508-000, Brazil*

**Contents**

1. ^1^H NMR spectra of RB-1......................................**S.I. 1**
2. ^13^C NMR spectra of RB-1.................................... **S.1. 2**
3. ^1^H NMR spectra of RB-2......................................**S.I. 3**
4. ^13^C NMR spectra of RB-2…..…............................**S.I. 4**
5. ^1^H NMR spectra of RB-3.......................................**S.I. 5**
6. ^13^C NMR spectra of RB-3…..…............................**S.I. 6**
7. FTIR spectra of chemosensor RB-1 with and without F ions ……**S.I.7**
8. FTIR spectra of chemosensor RB-2………………...S.I.8
9. FTIR spectra of chemosensor RB-3………………… S.I.9
10. Colour changes upon addition of F^-^ and CN^-^ ions ………. S.I.10
11. BH plot of chemosensor (**RB-1 to RB-3 Fluoride) S.I. 11-13**
12. Linear plot of chemosensor (RB-1 to RB-3 Fluoride)  **S.I. 14-16**
13. BH plot of chemosensor (**RB-1 to RB-3 Cyanide) S.I. 17-19**
14. Linear plot of chemosensor (RB-1 to RB-3 **Cyanide**) **S.I. 20-22**
15. Job`s plot for of chemosensor (**RB-1 to RB-3 Fluoride) S.I. 23-25**
16. Job`s plot for of chemosensor (**RB-1 to RB-3 Cyanide) S.I. 26-28**
17. **The color change of strips containing RB-1 to 3 with F^-^ ions S.I. 29**
18. AIM properties include Electronic density (ρ), Laplacian of density (∇^2^ ρ), ellipticity (ε) and density of potential energy (V) of chemosensor (**RB-1 to RB-3)** ……………………………………………………………………**S.I.30-32**


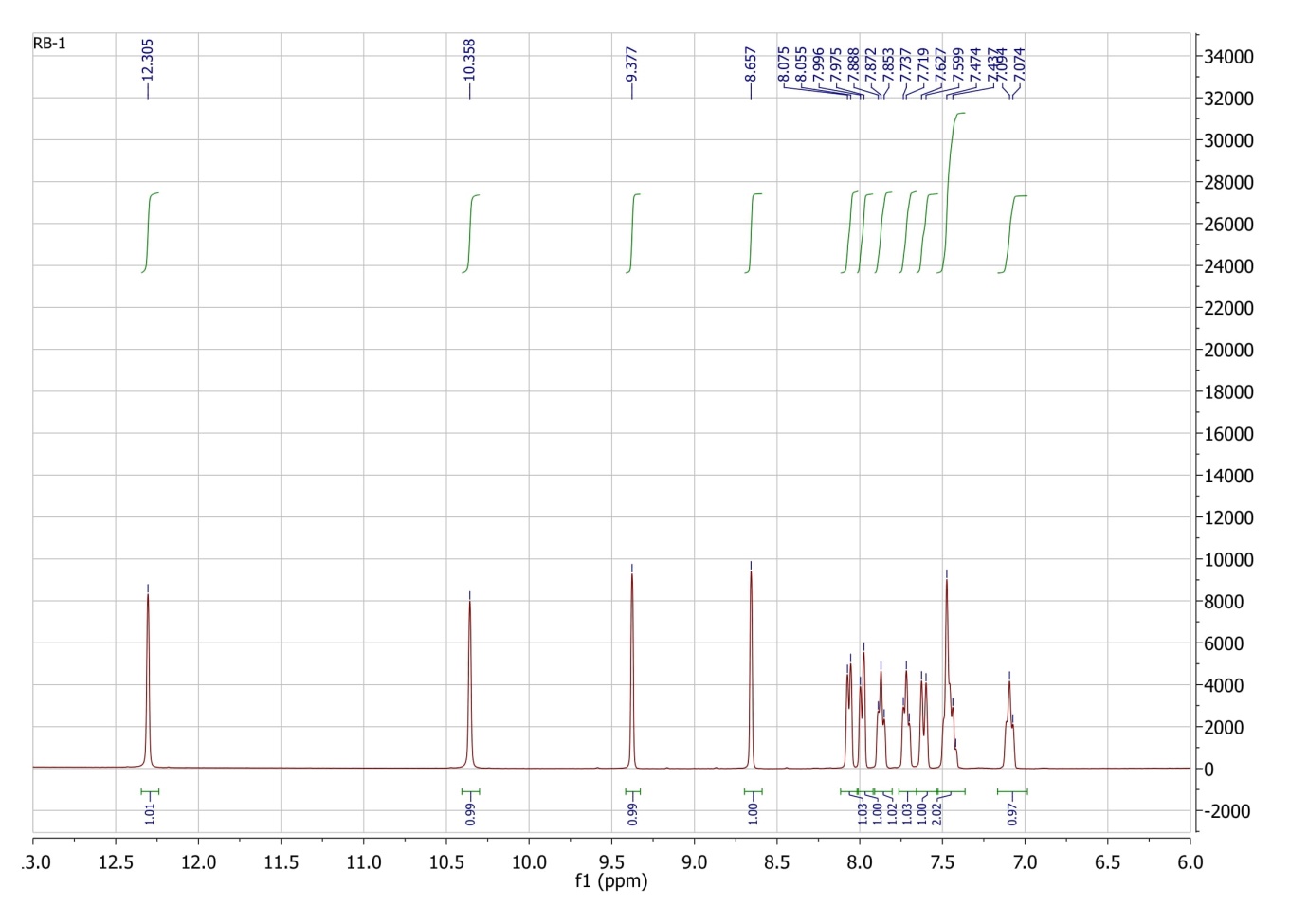


**S.1. 1** ^1^H NMR spectrum of RB-1 in DMSO d_6_.


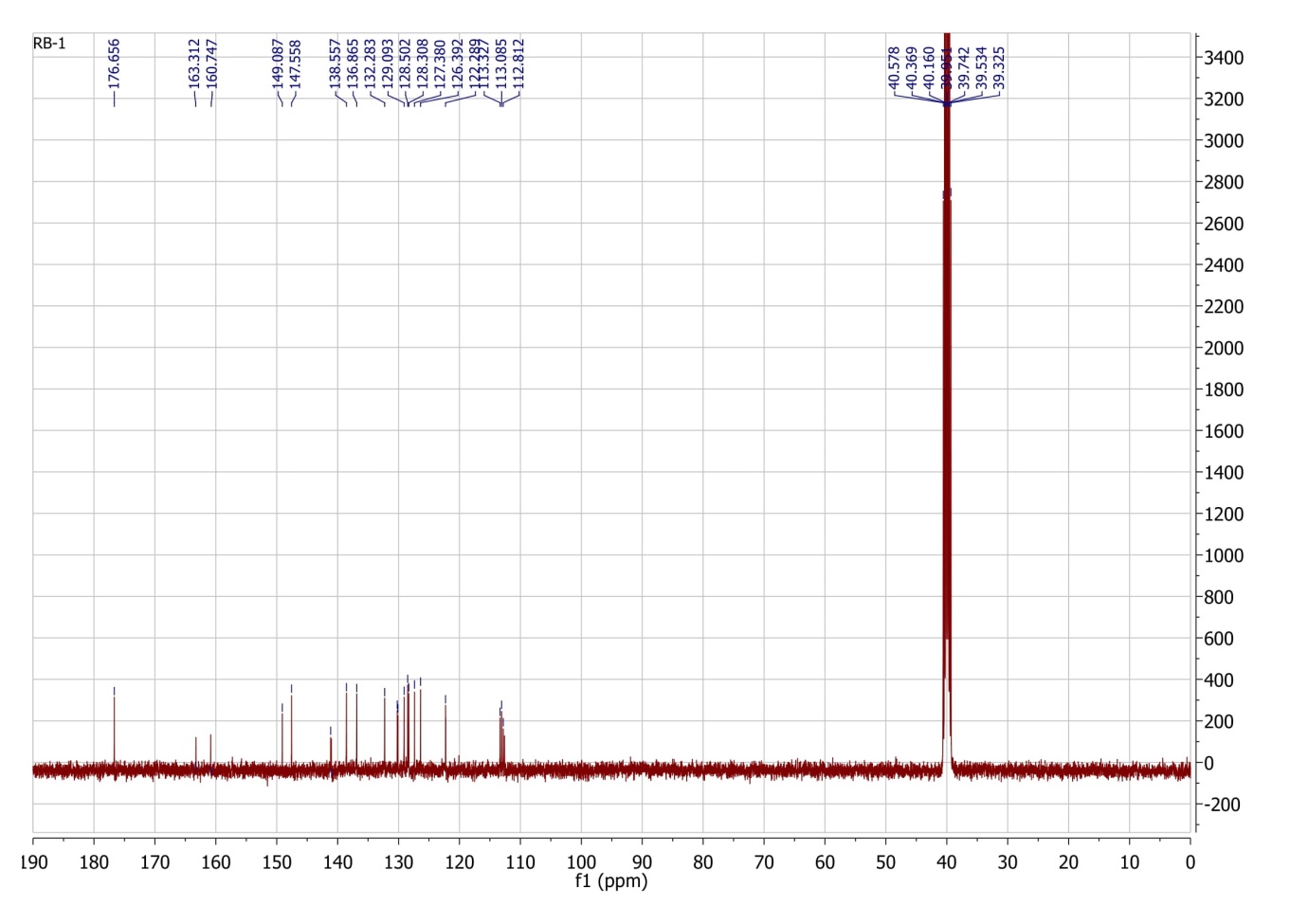


**S.1. 2 ^13^C NMR spectrum of RB-1 in DMSO d_6_.**


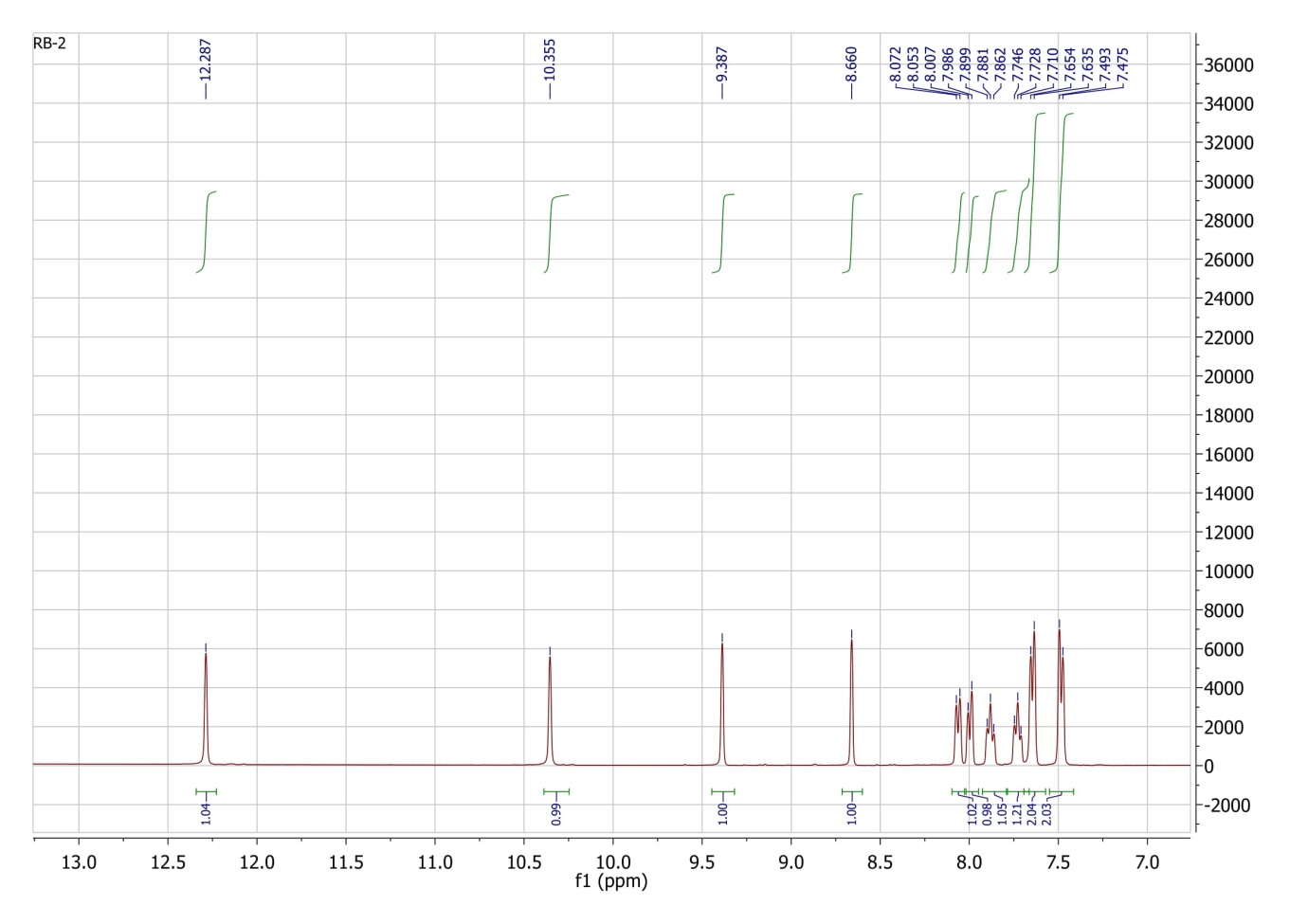


**S.1. 3 ^1^H NMR spectrum of RB-2 in DMSO d_6_.**


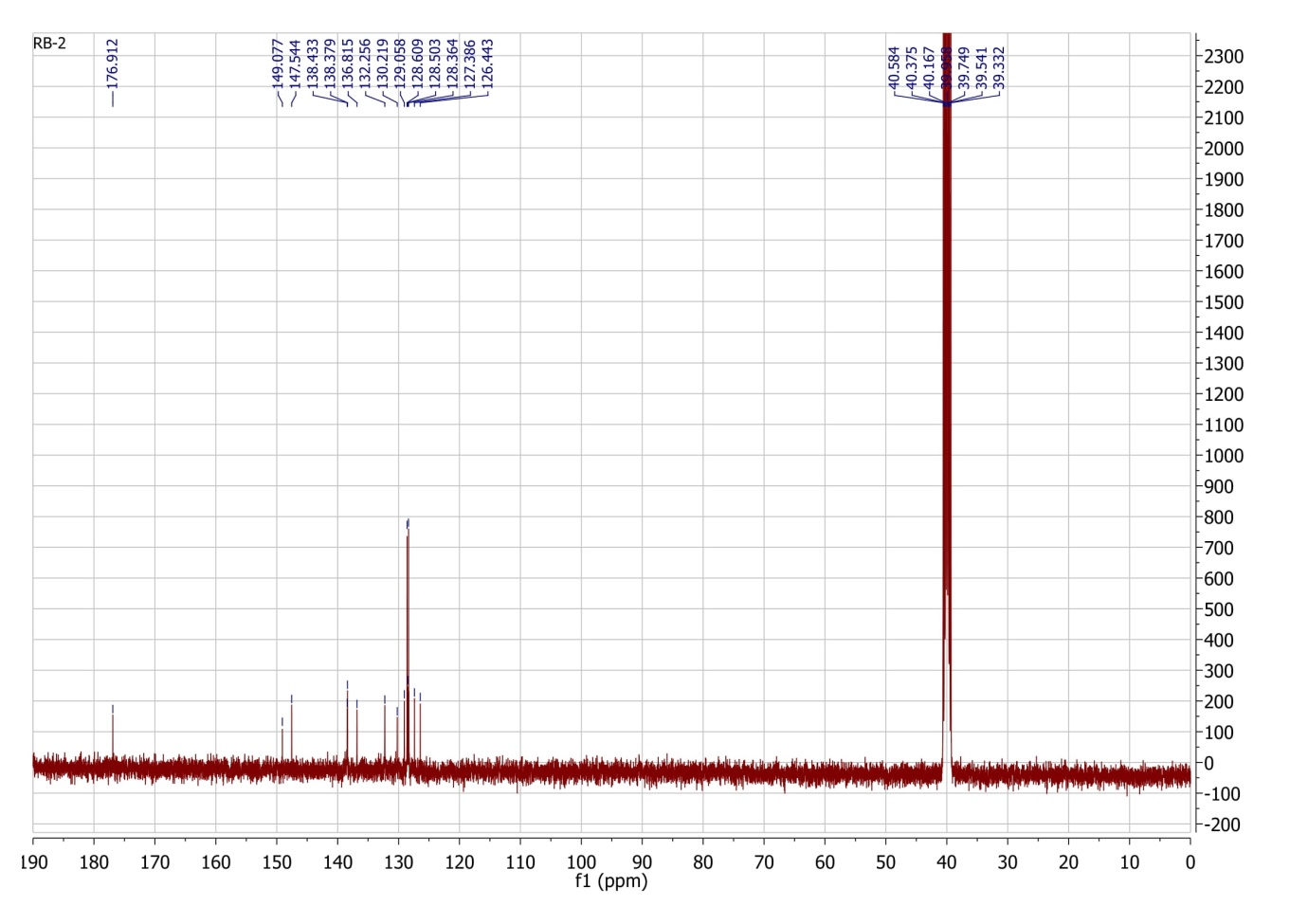


**S.1. 4 ^13^C NMR spectrum of RB-2 in DMSO d_6_.**


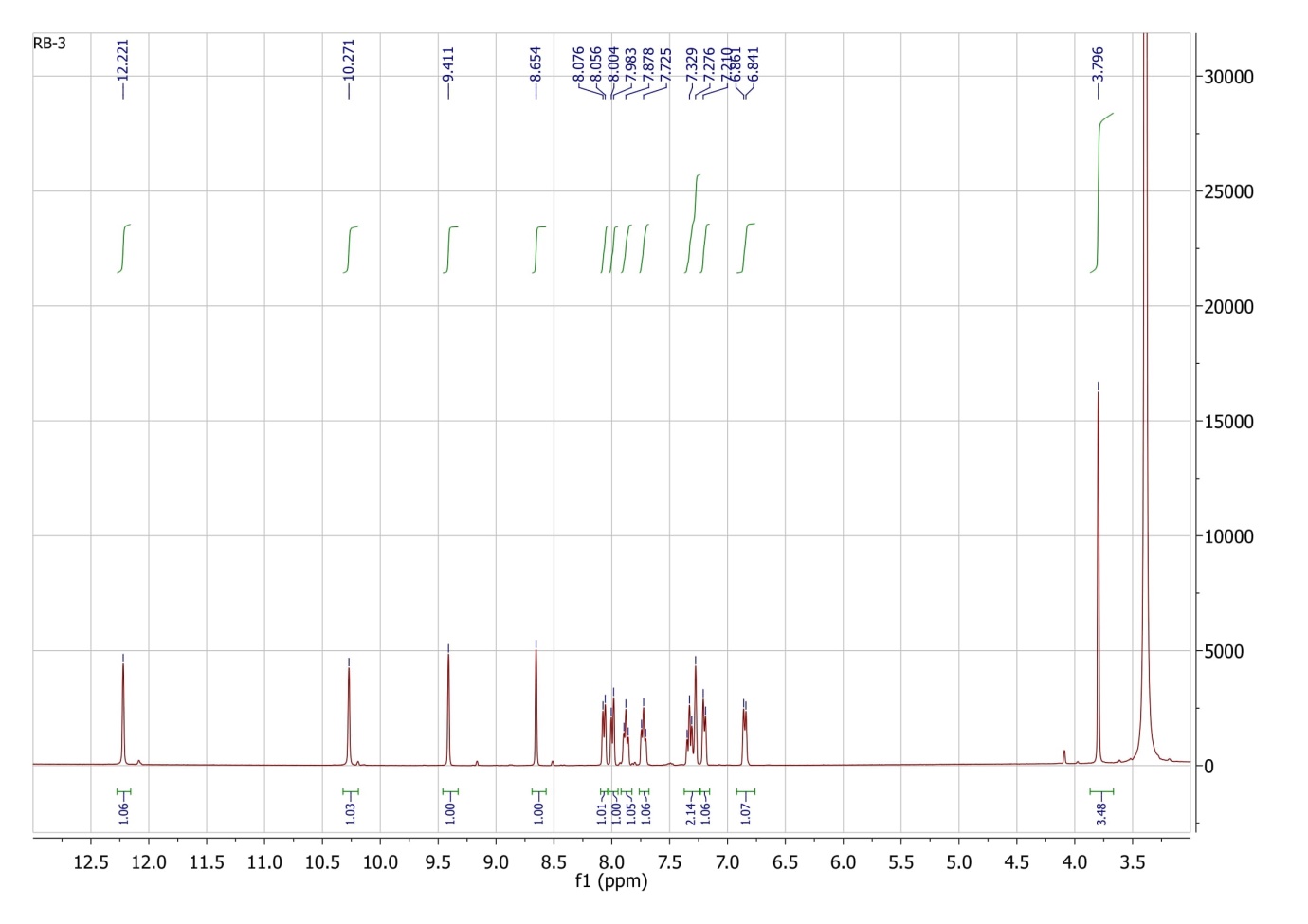


**S.1. 5 ^1^H NMR spectrum of RB-3 in DMSO d_6_.**

**
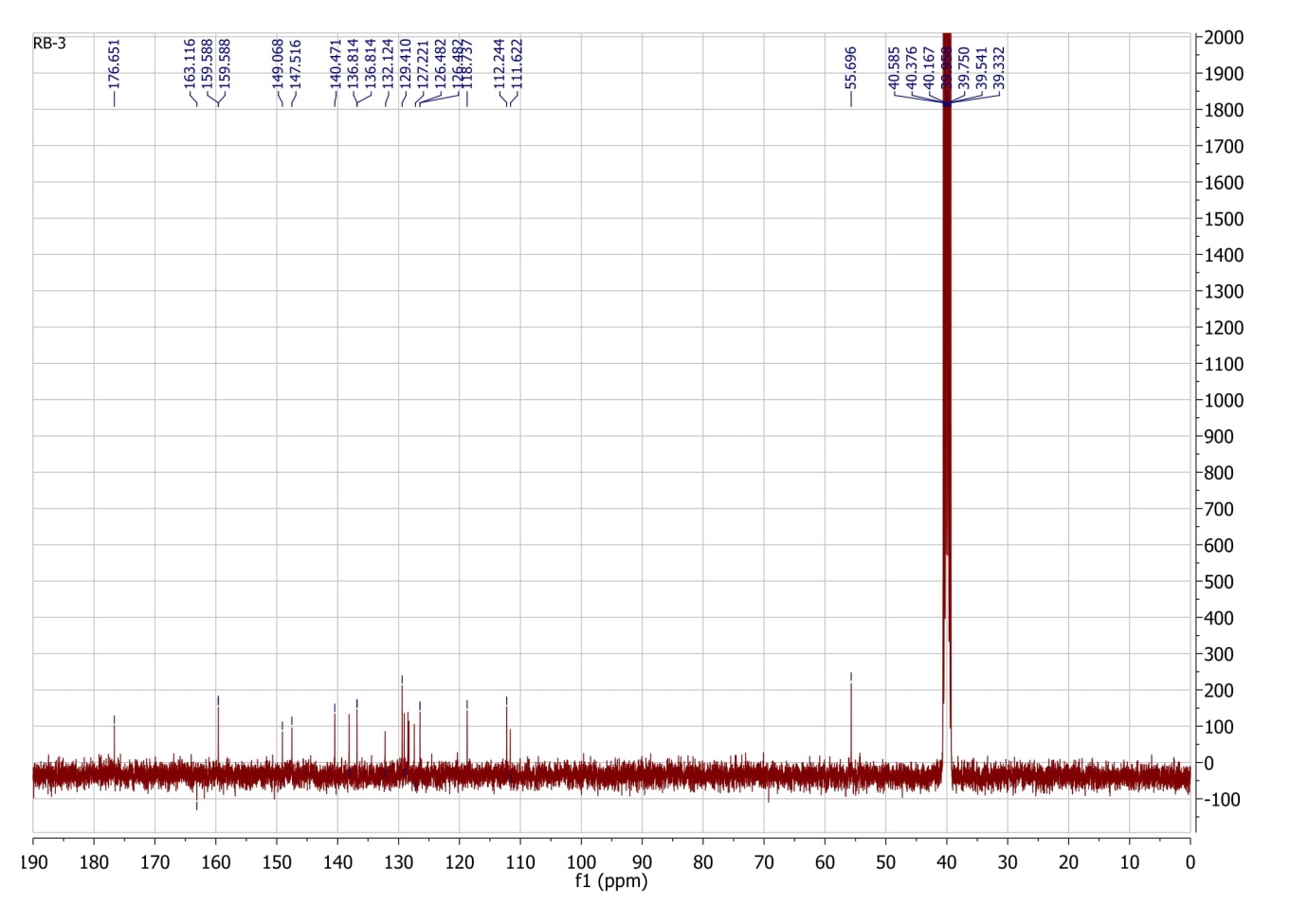
**

**S.1. 6 ^13^C NMR spectrum of RB-3 in DMSO d_6_.**

**
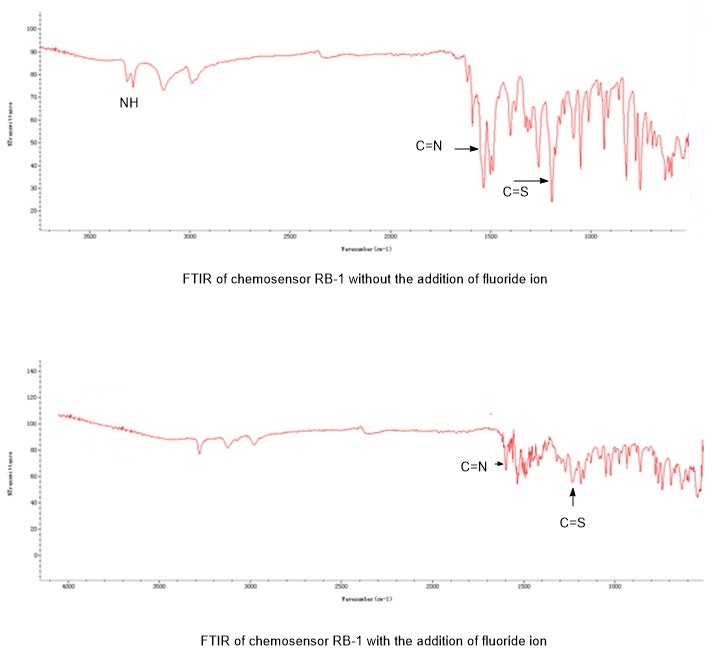
**

**S.I. 7. IR spectra of chemosensor RB-1 with and without the addition of F^-^ ions.**

**
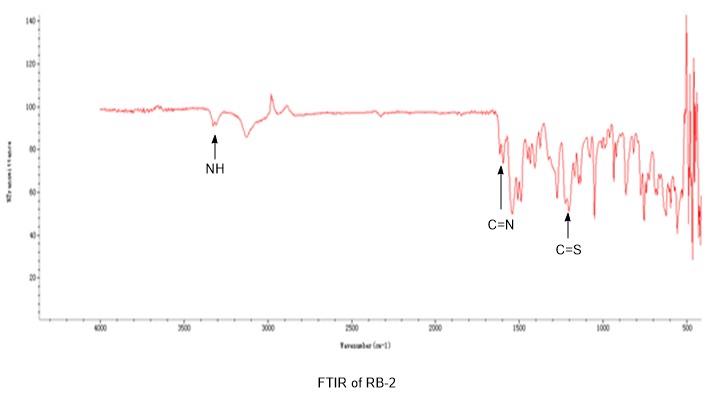
**

**S.I. 8. IR spectra of chemosensor RB-2.**

**
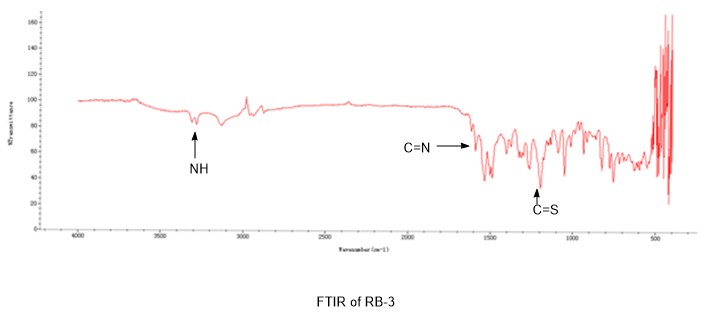
**

**S.I. 9. IR spectra of chemosensor RB-3.**

**
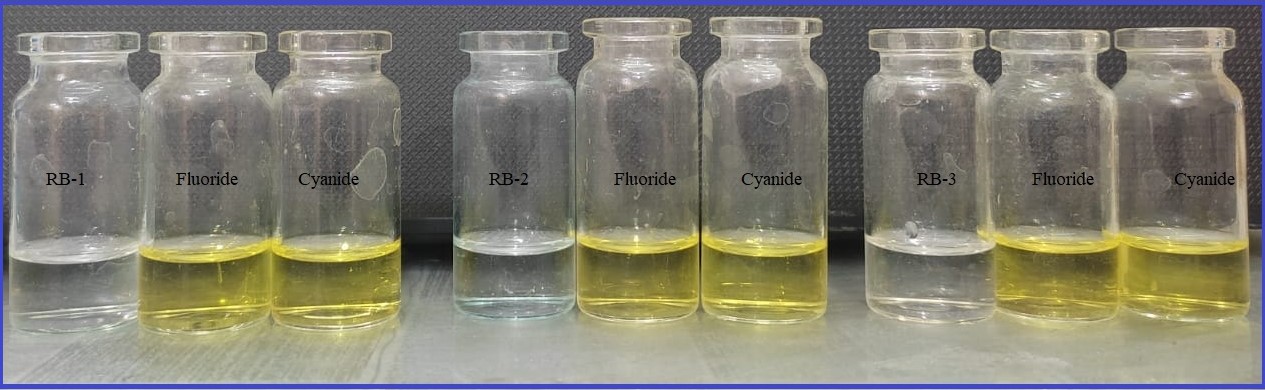
**

**S.I. 10**. The change in color of receptor RB-1-3 with anions





**S.I.11.** BH plot of chemosensor RB-1 (G= Fluoride)





**S.I. 12.** BH plot of chemosensor RB-2 (G= Fluoride)





**S.I. 13.** BH plot of chemosensor RB-3 (G= Fluoride)


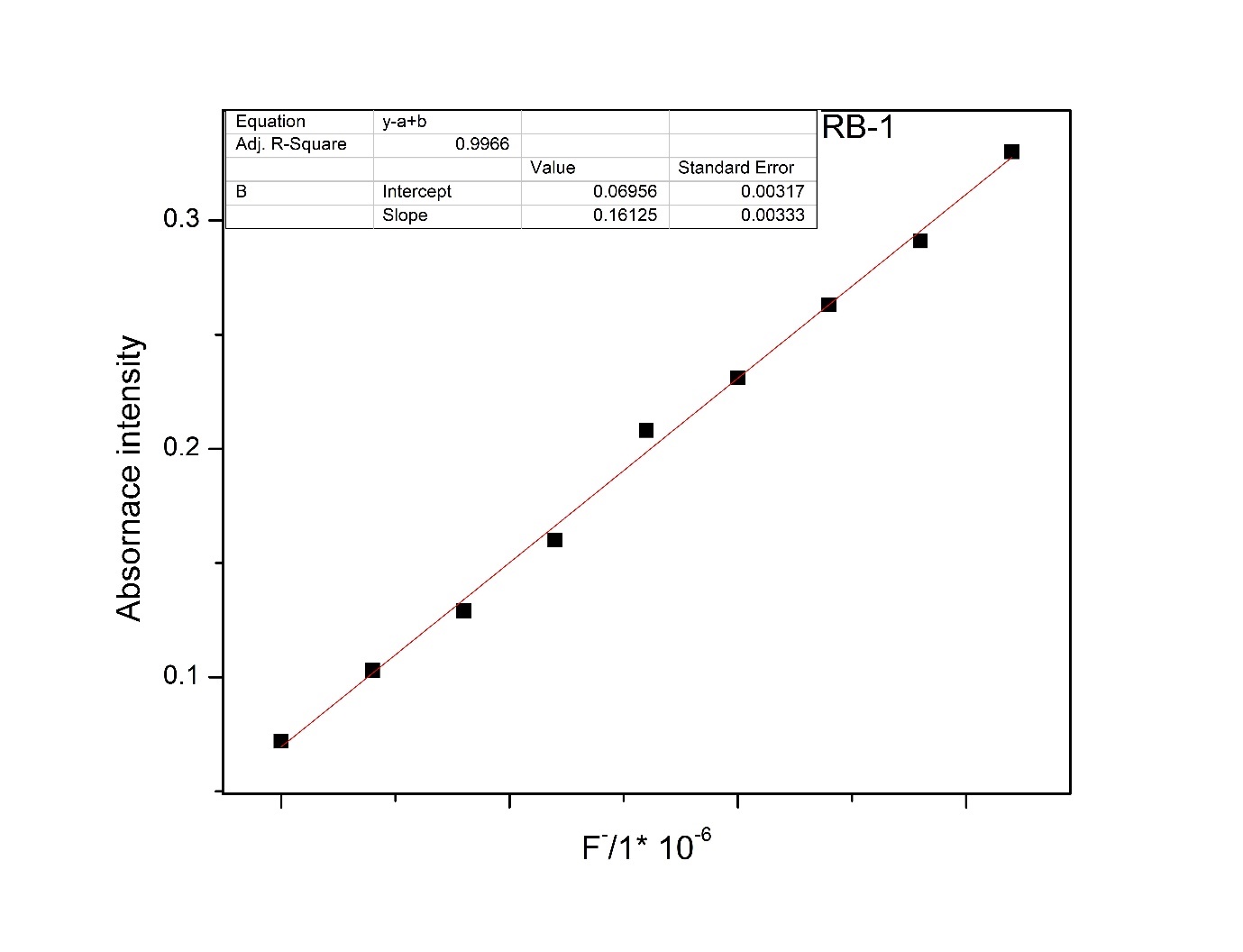


**S.I. 14.** Linear plot of chemosensor RB-1 (Fluoride)


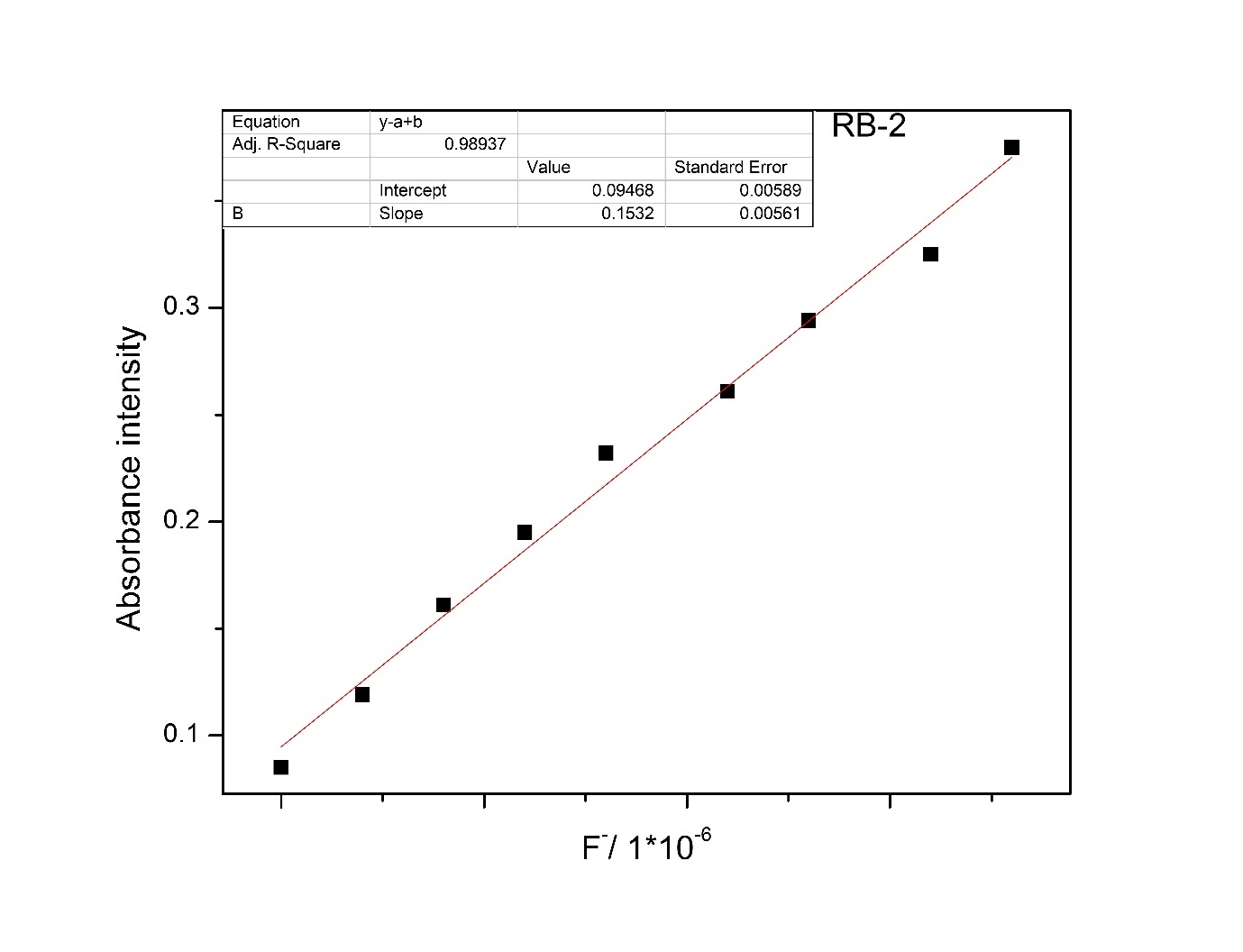


**S.I. 15.** Linear plot of chemosensor RB-2 (Fluoride)


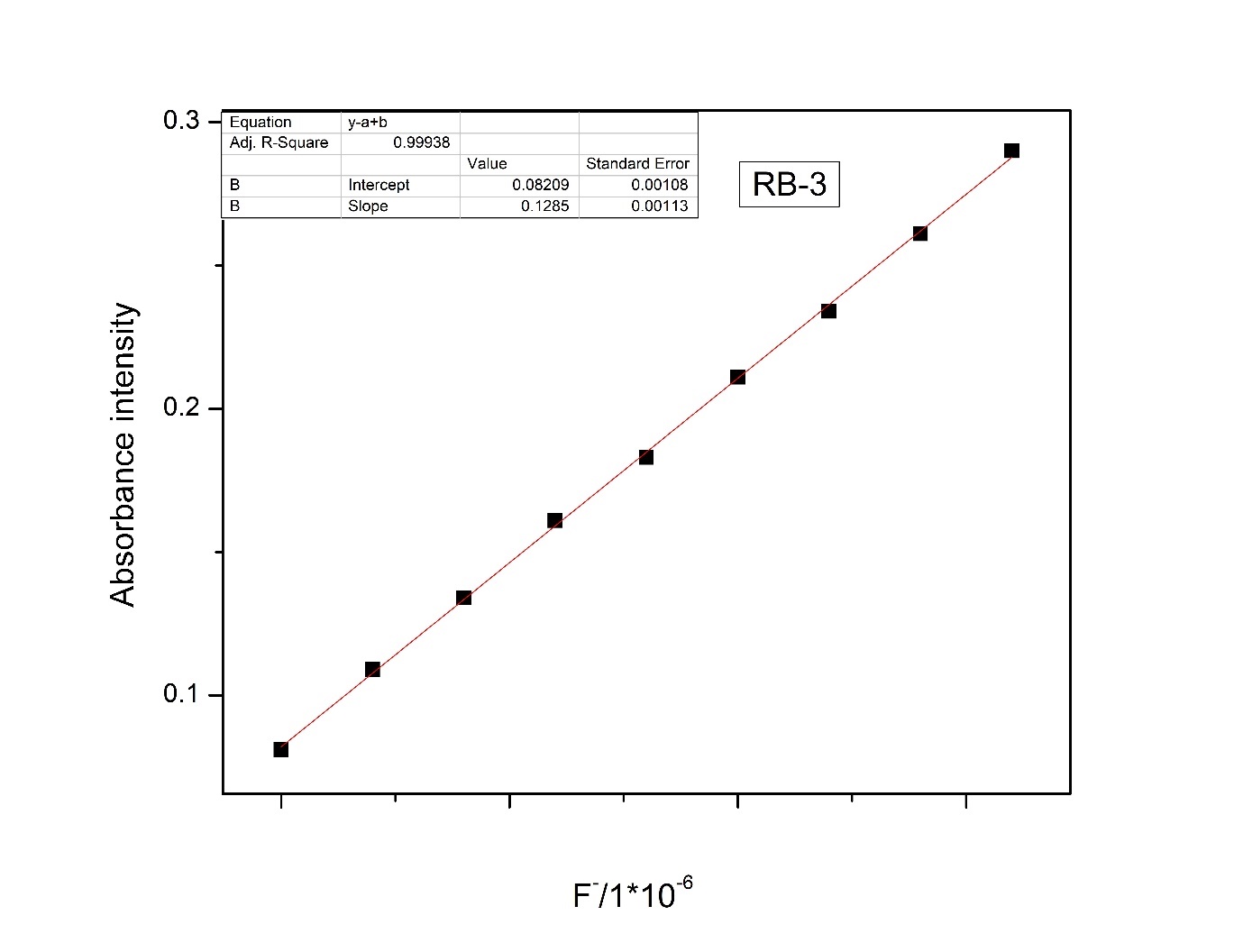


**S.I. 16.** Linear plot of chemosensor RB-3 (Fluoride)





**S.I. 17.** BH plot of chemosensor RB-1 (G= Cyanide)





**S.I. 18.** BH plot of chemosensor RB-2 (G= Cyanide)





**S.I. 19.** BH plot of chemosensor RB-3 (G= Cyanide)


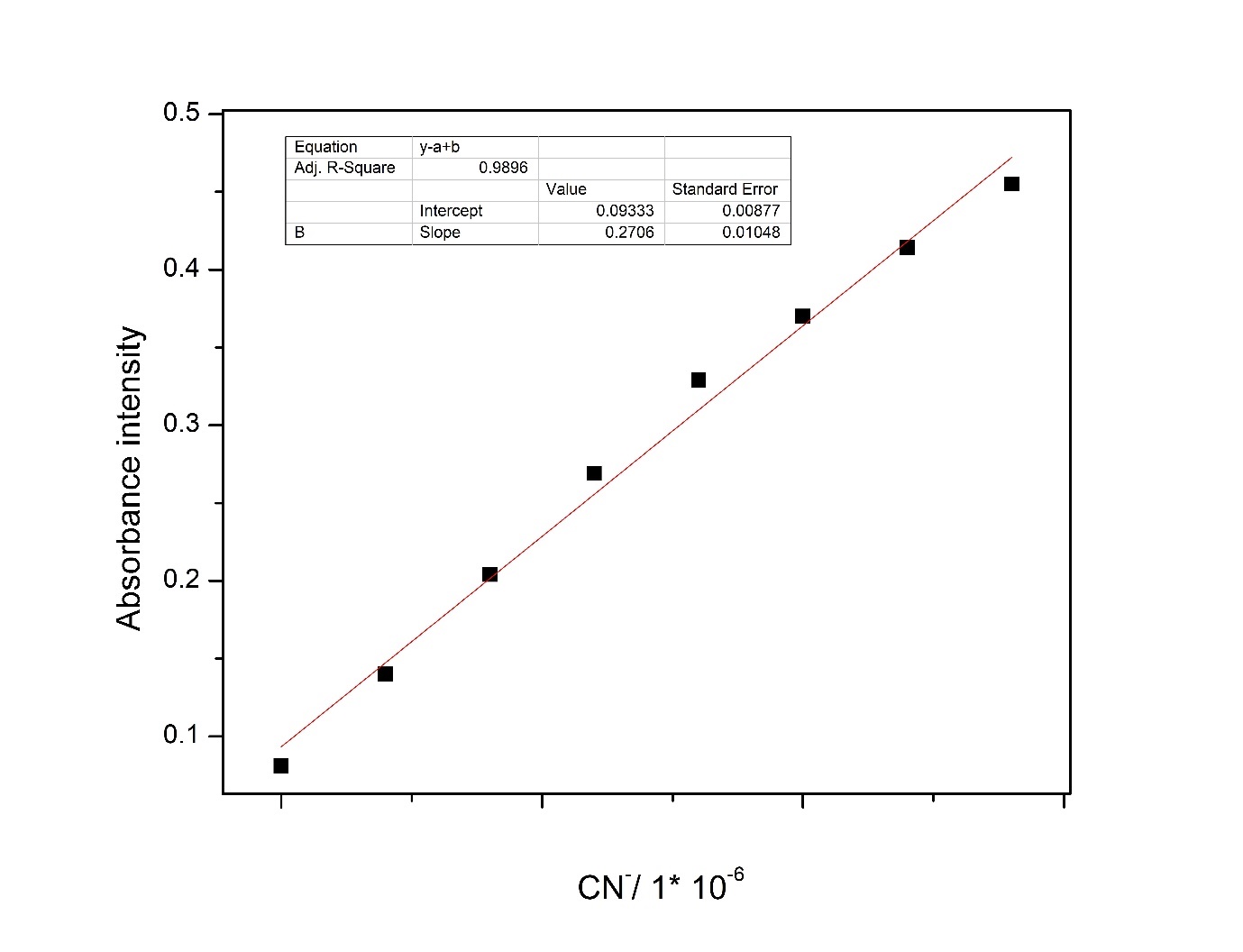


**S.I. 20.** Linear plot of chemosensor RB-1 (Cyanide)


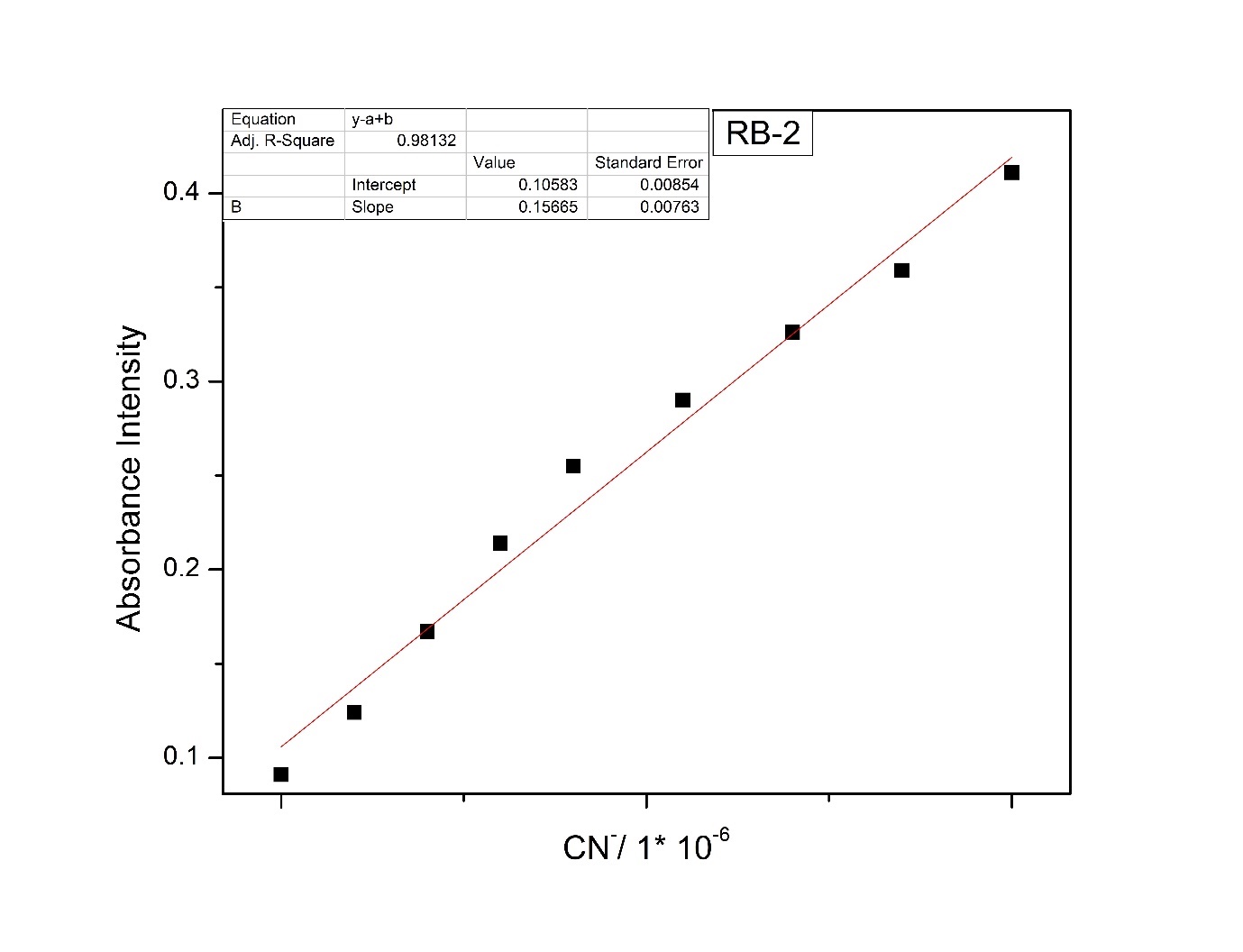


**S.I. 21.** Linear plot of chemosensor RB-2 (Cyanide)


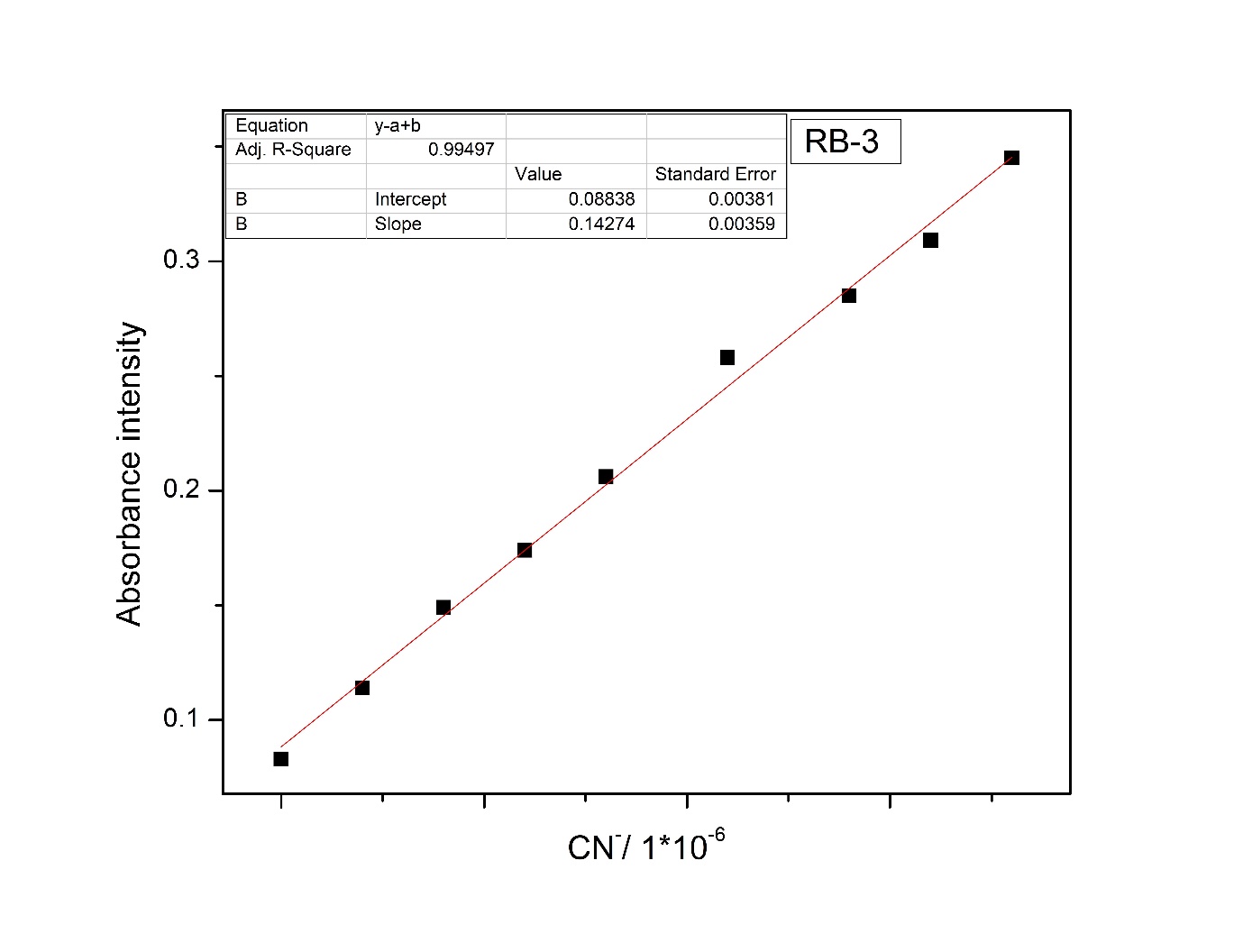


**S.I. 22.** Linear plot of chemosensor RB-3 (Cyanide)


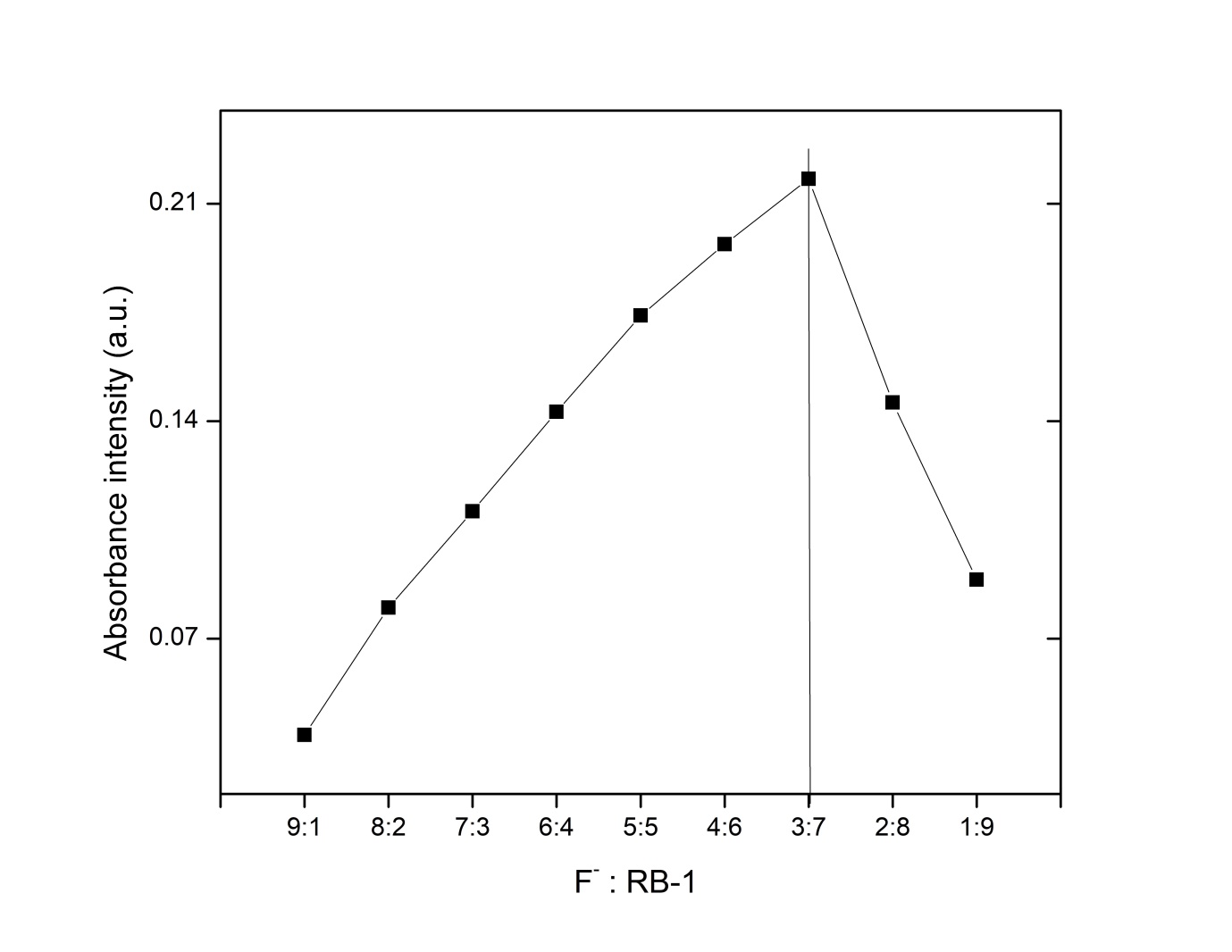


**S.I. 23.** Job`s Plot of chemosensor RB-1 (Fluoride)


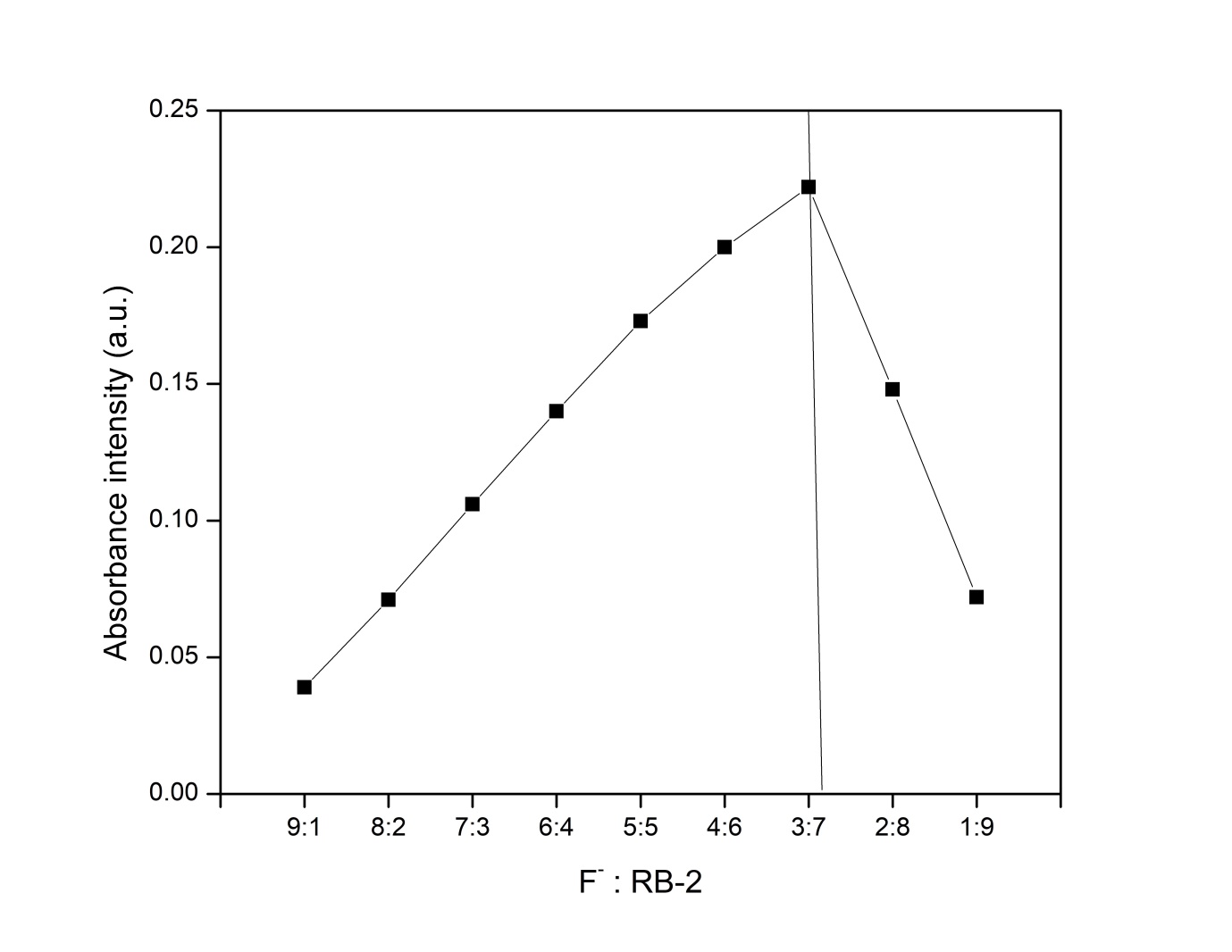


**S.I. 24.** Job`s Plot of chemosensor RB-2 (Fluoride)


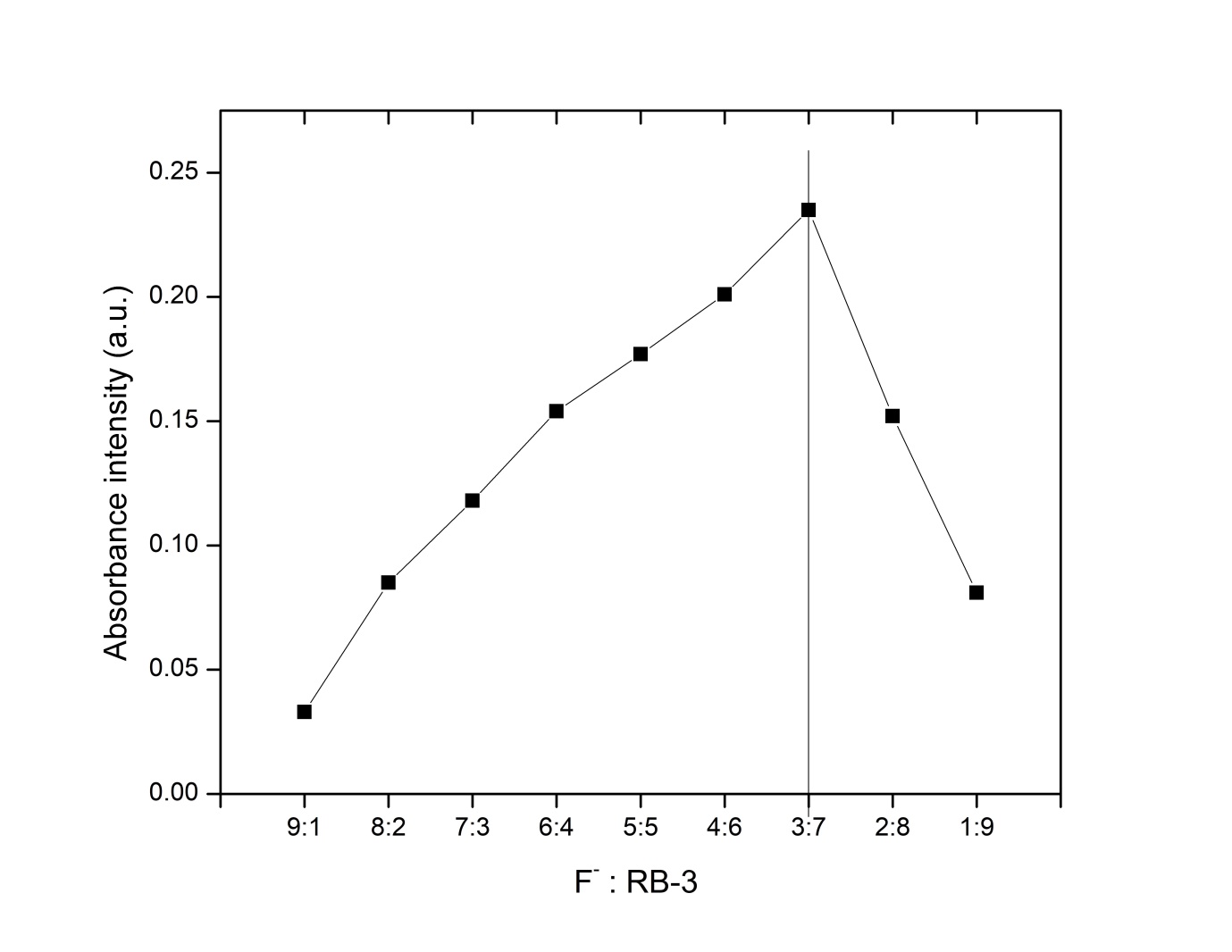


**S.I. 25.** Job`s Plot of chemosensor RB-3 (Fluoride)


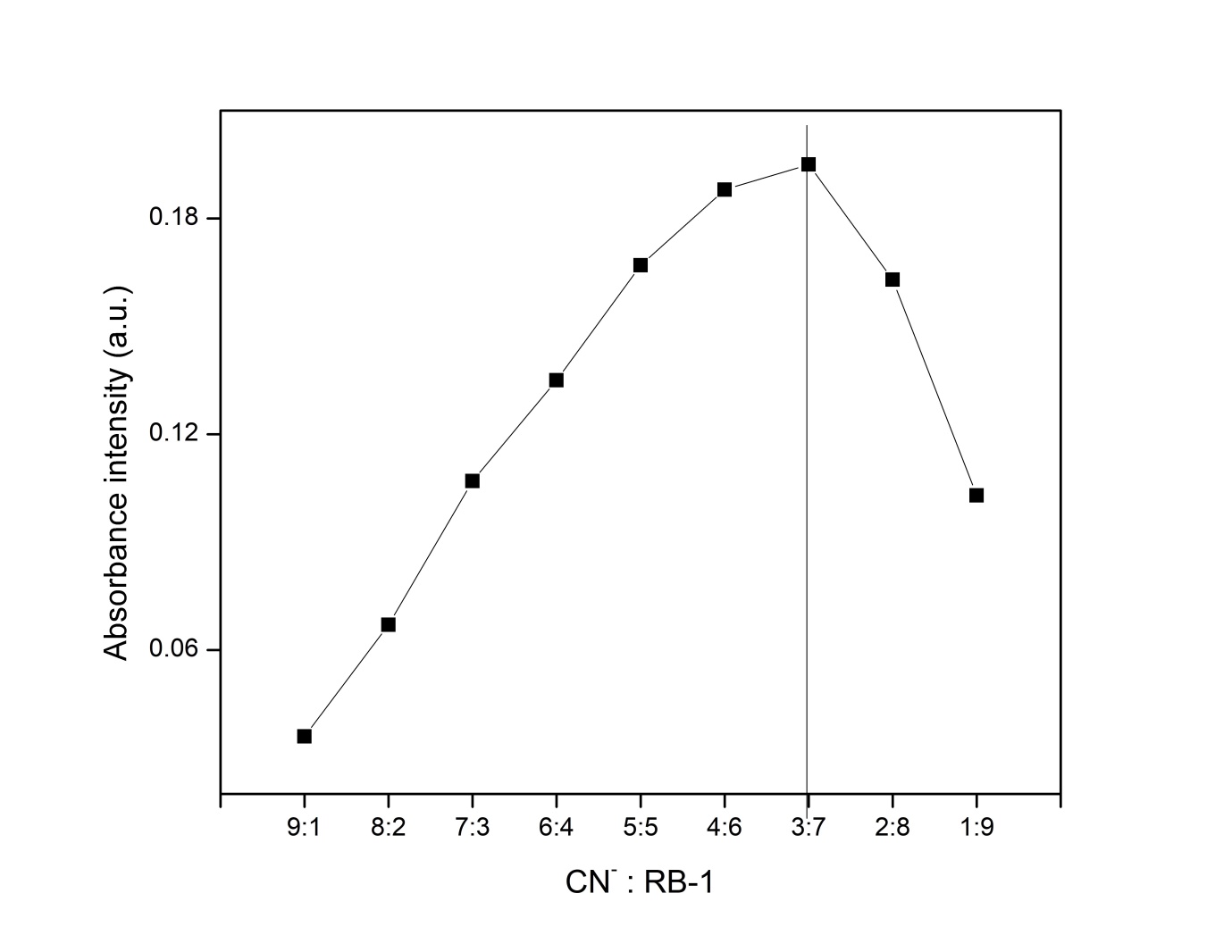


**S.I. 26.** Job`s Plot of chemosensor RB-1 (Cyanide)


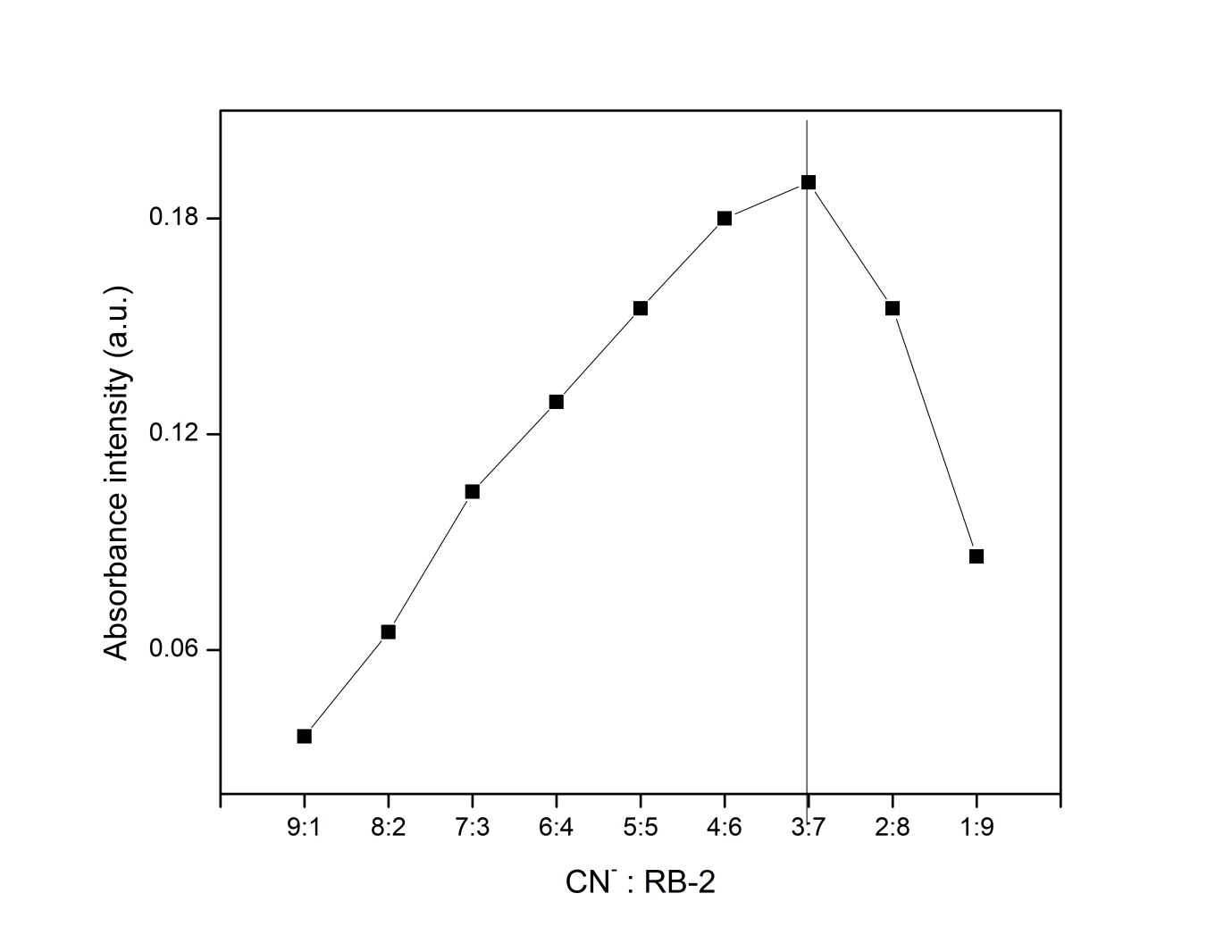


**S.I. 27.** Job`s Plot of chemosensor RB-2 (Cyanide)


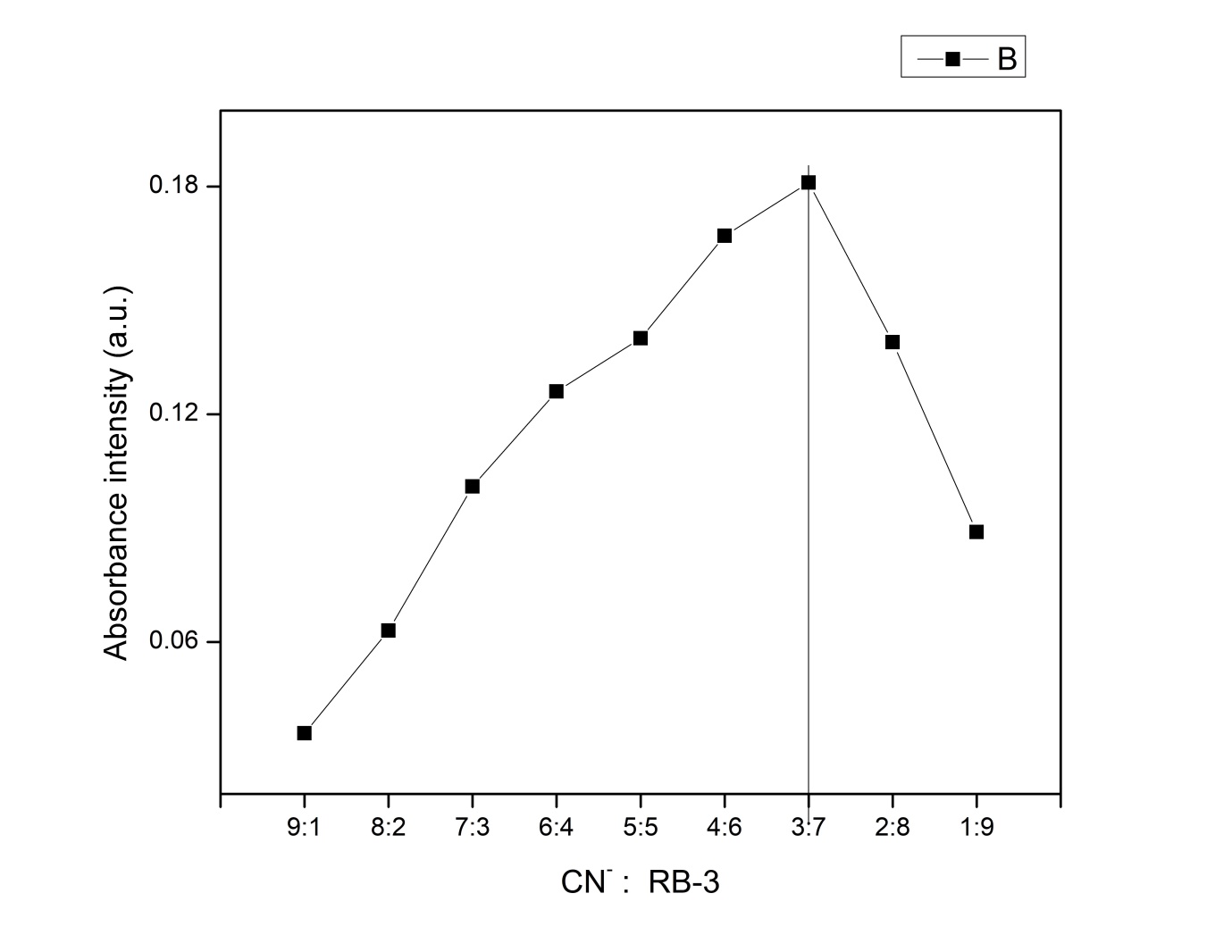


**S.I. 28.** Job`s Plot of chemosensor RB-3 (Cyanide)


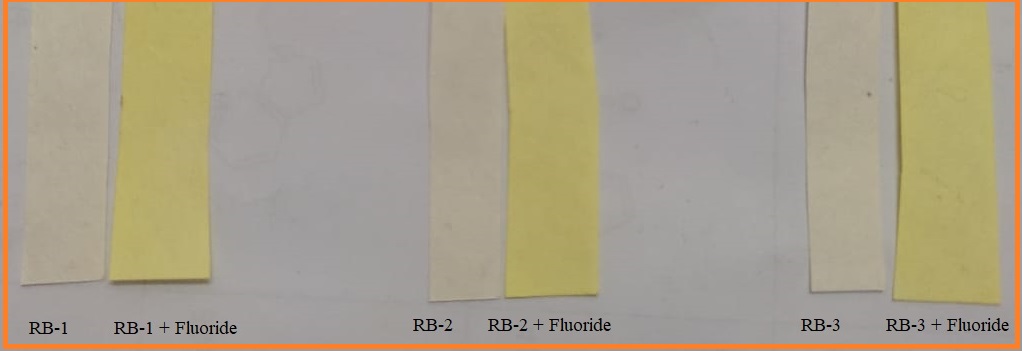


**S.I. 29. The color change of strips containing RB-1 to 3 with F^-^ ions.**

**Table S.I. 30:** AIM properties include Electronic density (ρ), Laplacian of density (∇^2^ ρ), ellipticity (ε) and density of potential energy (V) of **RB-1** compound.

| **BCP** | **Bond** | $\boldsymbol{\rho}$ **(e/a^3^)** | $\boldsymbol{\nabla}^{\boldsymbol{2}}\boldsymbol{\rho}$ **(e/a^5^)** | $\boldsymbol{\varepsilon}$ | $\boldsymbol{V}$ **(hartree.e/a^3^)** |
| --- | --- | --- | --- | --- | --- |
| 1 | C1 - C2 | +0.300858 | -0.826486 | +0.206764 | -0.373396 |
| 2 | C3 - C4 | +0.318759 | -0.829985 | +0.507697 | -0.450887 |
| 3 | C7 - C8 | +0.318044 | -0.805024 | +0.517348 | -0.449886 |
| 4 | C2 - C3 | +0.305790 | -0.952944 | +0.021596 | -0.358750 |
| 5 | C4 - C5 | +0.299310 | -0.907147 | +0.023775 | -0.347849 |
| 6 | C1 - C6 | +0.300054 | -0.815085 | +0.182639 | -0.372746 |
| 7 | C4 - H26 | +0.286851 | -0.993447 | +0.020943 | -0.336310 |
| 8 | C5 - C6 | +0.319129 | -0.830650 | +0.510377 | -0.452435 |
| 9 | C1 - C7 | +0.305353 | -0.841142 | +0.184790 | -0.386820 |
| 10 | C5 - H27 | +0.286502 | -0.990029 | +0.023678 | -0.336201 |
| 11 | C3 - H25 | +0.287170 | -1.002242 | +0.023205 | -0.336574 |
| 12 | C8 - C12 | +0.282866 | -0.797375 | +0.025926 | -0.308702 |
| 13 | C2 - N10 | +0.326564 | -1.010842 | +0.442083 | -0.713883 |
| 14 | C8 - C9 | +0.292357 | -0.896854 | +0.010308 | -0.330568 |
| 15 | C9 - N10 | +0.370070 | -1.672098 | +0.274768 | -0.809618 |
| 16 | C9 - Cl11 | +0.191214 | -0.361873 | +0.093637 | -0.163252 |
| 17 | C12 - N13 | +0.360279 | -0.431383 | +0.402065 | -1.088009 |
| 18 | C12 - H30 | +0.286601 | -1.042904 | +0.018253 | -0.326583 |
| 19 | C7 - H29 | +0.286842 | -1.011486 | +0.010684 | -0.334290 |
| 20 | N13 - N14 | +0.351706 | -0.758659 | +0.090524 | -0.486213 |
| 21 | C15 - S17 | +0.197465 | -0.430594 | +0.130084 | -0.205012 |
| 22 | N14 - C15 | +0.365494 | -1.094025 | +0.377079 | -0.941217 |
| 23 | C15 - N16 | +0.327247 | -1.306309 | +0.057799 | -0.617711 |
| 24 | N16 - C18 | +0.280712 | -0.925100 | +0.047705 | -0.510563 |
| 25 | N16 - H32 | +0.347767 | -1.803200 | +0.060603 | -0.572325 |
| 26 | C18 - C19 | +0.310330 | -0.773079 | +0.528873 | -0.426331 |
| 27 | C19 - C20 | +0.311482 | -0.992560 | +0.029547 | -0.378656 |
| 28 | C18 - C23 | +0.309041 | -0.976090 | +0.046504 | -0.377059 |
| 29 | N14 - H36 | +0.011203 | +0.044204 | +0.347936 | -0.008006 |
| 30 | C20 - C21 | +0.319303 | -0.846066 | +0.567971 | -0.450584 |
| 31 | C22 - C23 | +0.311939 | -0.785261 | +0.494818 | -0.429915 |
| 32 | C21 - F24 | +0.254070 | +0.397651 | +0.029864 | -0.793757 |
| 33 | C21 - C22 | +0.319567 | -1.066704 | +0.066553 | -0.400374 |
| 34 | C23 - H36 | +0.291299 | -1.035456 | +0.020985 | -0.344404 |
| 35 | C6 - H28 | +0.284702 | -0.979623 | +0.025088 | -0.333510 |
| 36 | H30 - F37 | +0.011702 | +0.059337 | +0.037042 | -0.013336 |
| 37 | N14 - F37 | +0.007210 | +0.062415 | +2.473273 | -0.014481 |
| 38 | N14 - H31 | +0.007573 | +0.045795 | +0.067681 | -0.035477 |
| 39 | H36 - F37 | +0.009453 | +0.051447 | +0.344924 | -0.010347 |
| 40 | C19 - H33 | +0.284081 | -0.970897 | +0.023984 | -0.332961 |
| 41 | C20 - H34 | +0.286901 | -0.996095 | +0.025214 | -0.337296 |
| 42 | C22 - H35 | +0.287910 | -1.007112 | +0.022836 | -0.337874 |
| 43 | H31 - F37 | +0.020995 | -0.250776 | +0.003027 | -0.402923 |

**Table S.I. 31:** AIM properties include Electronic density (ρ), Laplacian of density (∇^2^ ρ), ellipticity (ε) and density of potential energy (V) of **RB2** compound.

| **BCP** | **Bond** | $\boldsymbol{\rho}$ **(e/a^3^)** | $\boldsymbol{\nabla}^{\boldsymbol{2}}\boldsymbol{\rho}$ **(e/a^5^)** | $\boldsymbol{\varepsilon}$ | $\boldsymbol{V}$ **(hartree.e/a^3^)** |
| --- | --- | --- | --- | --- | --- |
| 1 | C1 - C2 | +0.300792 | -0.768598 | +0.348236 | -0.387577 |
| 2 | C3 - C4 | +0.318737 | -0.829681 | +0.508069 | -0.450847 |
| 3 | C7 - C8 | +0.318082 | -0.805405 | +0.516814 | -0.449937 |
| 4 | C2 - C3 | +0.305776 | -0.947795 | +0.029645 | -0.360037 |
| 5 | C4 - C5 | +0.299260 | -0.906793 | +0.023688 | -0.347719 |
| 6 | C1 - C6 | +0.300193 | -0.855465 | +0.102753 | -0.363189 |
| 7 | C4 - H25 | +0.286905 | -0.993967 | +0.020827 | -0.336344 |
| 8 | C5 - C6 | +0.319216 | -0.831522 | +0.509030 | -0.452550 |
| 9 | C1 - C7 | +0.305646 | -0.887011 | +0.097811 | -0.376469 |
| 10 | C5 - H26 | +0.286549 | -0.990465 | +0.023668 | -0.336256 |
| 11 | C8 - C12 | +0.282762 | -0.796417 | +0.026534 | -0.308586 |
| 12 | C2 - N10 | +0.326515 | -1.117264 | +0.194015 | -0.686911 |
| 13 | C8 - C9 | +0.292390 | -0.881883 | +0.035533 | -0.334405 |
| 14 | C3 - H24 | +0.287183 | -1.002250 | +0.023425 | -0.336600 |
| 15 | C9 - N10 | +0.369973 | -1.381842 | +0.043588 | -0.881312 |
| 16 | C12 - H29 | +0.286608 | -1.043631 | +0.017702 | -0.326505 |
| 17 | C9 - Cl11 | +0.191332 | -0.343357 | +0.037811 | -0.168128 |
| 18 | C12 - N13 | +0.360432 | -0.436419 | +0.397485 | -1.087739 |
| 19 | C7 - H28 | +0.286964 | -1.012908 | +0.009859 | -0.334410 |
| 20 | N13 - N14 | +0.351168 | -0.755538 | +0.090943 | -0.485255 |
| 21 | N14 - C15 | +0.365520 | -1.080077 | +0.386264 | -0.946834 |
| 22 | C15 - N16 | +0.324200 | -1.274741 | +0.078380 | -0.634767 |
| 23 | N16 - C18 | +0.281288 | -0.920127 | +0.028192 | -0.541101 |
| 24 | C15 - S17 | +0.197704 | -0.431775 | +0.132808 | -0.206151 |
| 25 | C18 - C19 | +0.310322 | -0.774150 | +0.528363 | -0.427094 |
| 26 | C19 - C20 | +0.312544 | -1.002073 | +0.025730 | -0.380362 |
| 27 | C18 - C23 | +0.308978 | -0.978015 | +0.049532 | -0.379083 |
| 28 | N14 - H35 | +0.011622 | +0.045593 | +0.290530 | -0.008489 |
| 29 | C20 - C21 | +0.311529 | -0.786041 | +0.506236 | -0.431661 |
| 30 | C22 - C23 | +0.312095 | -0.783109 | +0.500403 | -0.430516 |
| 31 | C21 - Cl36 | +0.193491 | -0.319627 | +0.041655 | -0.181444 |
| 32 | C21 - C22 | +0.311802 | -0.992996 | +0.039601 | -0.383721 |
| 33 | C23 - H35 | +0.291494 | -1.033147 | +0.026203 | -0.345857 |
| 34 | C6 - H27 | +0.284726 | -0.980276 | +0.024431 | -0.333414 |
| 35 | H29 - F37 | +0.011652 | +0.059160 | +0.027701 | -0.013255 |
| 36 | N14 - H30 | +0.007589 | +0.045589 | +0.067930 | -0.035414 |
| 37 | N14 - F37 | +0.007226 | +0.062512 | +2.459729 | -0.014478 |
| 38 | H35 - F37 | +0.009795 | +0.052464 | +0.286097 | -0.011042 |
| 39 | N16 - H31 | +0.197334 | -0.622117 | +0.038909 | -0.592606 |
| 40 | C19 - H32 | +0.283886 | -0.966493 | +0.027541 | -0.333314 |
| 41 | C20 - H33 | +0.287987 | -1.013577 | +0.019892 | -0.336923 |
| 42 | C22 - H34 | +0.288862 | -1.018154 | +0.024624 | -0.338537 |
| 43 | H30 - F37 | +0.021013 | -0.236888 | +0.002879 | -0.403565 |

**Table S.I. 32:** AIM properties include Electronic density (ρ), Laplacian of density (∇^2^ ρ), ellipticity (ε) and density of potential energy (V) of **RB3** compound.

| **BCP** | **Bond** | $\boldsymbol{\rho}$ **(e/a^3^)** | $\boldsymbol{\nabla}^{\boldsymbol{2}}\boldsymbol{\rho}$ **(e/a^5^)** | $\boldsymbol{\varepsilon}$ | $\boldsymbol{V}$ **(hartree.e/a^3^)** |
| --- | --- | --- | --- | --- | --- |
| 1 | C1 - C2 | +0.300687 | -0.767986 | +0.348424 | -0.387409 |
| 2 | C3 - C4 | +0.318903 | -0.830819 | +0.508133 | -0.451317 |
| 3 | C2 - C3 | +0.305796 | -0.947768 | +0.029594 | -0.359992 |
| 4 | C4 - C5 | +0.299500 | -0.908461 | +0.023898 | -0.348301 |
| 5 | C7 - C8 | +0.318155 | -0.805675 | +0.517257 | -0.450150 |
| 6 | C1 - C6 | +0.299704 | -0.852514 | +0.102675 | -0.362083 |
| 7 | C4 - H25 | +0.286774 | -0.992645 | +0.021071 | -0.336242 |
| 8 | C1 - C7 | +0.304872 | -0.882321 | +0.097546 | -0.374656 |
| 9 | C5 - H26 | +0.286455 | -0.989483 | +0.023753 | -0.336166 |
| 10 | C5 - C6 | +0.318997 | -0.830340 | +0.508911 | -0.451954 |
| 11 | C8 - C12 | +0.282841 | -0.796953 | +0.026010 | -0.308627 |
| 12 | C2 - N10 | +0.326633 | -1.117801 | +0.194261 | -0.687255 |
| 13 | C12 - N13 | +0.360257 | -0.432248 | +0.402267 | -1.087760 |
| 14 | C8 - C9 | +0.292267 | -0.881319 | +0.035076 | -0.334211 |
| 15 | C3 - H24 | +0.287179 | -1.002166 | +0.023276 | -0.336679 |
| 16 | C9 - N10 | +0.370330 | -1.378912 | +0.043239 | -0.884924 |
| 17 | C12 - H29 | +0.286921 | -1.046026 | +0.018479 | -0.327366 |
| 18 | C9 - Cl11 | +0.191034 | -0.342244 | +0.037940 | -0.167730 |
| 19 | C7 - H28 | +0.286718 | -1.010979 | +0.010332 | -0.333895 |
| 20 | N13 - N14 | +0.351779 | -0.759686 | +0.090661 | -0.486196 |
| 21 | N14 - C15 | +0.365718 | -1.090598 | +0.385859 | -0.944387 |
| 22 | C15 - N16 | +0.325344 | -1.283194 | +0.077003 | -0.635254 |
| 23 | N16 - C18 | +0.280089 | -0.927170 | +0.002102 | -0.532734 |
| 24 | C15 - S17 | +0.197434 | -0.430105 | +0.130200 | -0.204557 |
| 25 | C19 - C20 | +0.313809 | -1.013489 | +0.056857 | -0.384318 |
| 26 | C18 - C19 | +0.312675 | -0.792357 | +0.546036 | -0.437663 |
| 27 | C20 - O36 | +0.288413 | -0.337004 | +0.037932 | -0.798355 |
| 28 | C18 - C23 | +0.307811 | -0.972212 | +0.051167 | -0.378481 |
| 29 | C20 - C21 | +0.309465 | -0.786699 | +0.560567 | -0.432702 |
| 30 | N14 - H34 | +0.011540 | +0.045289 | +0.323849 | -0.008298 |
| 31 | C22 - C23 | +0.312242 | -0.786810 | +0.493314 | -0.431436 |
| 32 | C21 - H35 | +0.286021 | -0.979667 | +0.040275 | -0.340279 |
| 33 | C21 - C22 | +0.309109 | -0.971890 | +0.032152 | -0.372683 |
| 34 | C23 - H34 | +0.290094 | -1.017663 | +0.034579 | -0.345648 |
| 35 | C6 - H27 | +0.284678 | -0.979950 | +0.024382 | -0.333338 |
| 36 | H29 - F41 | +0.012629 | +0.063251 | +0.031132 | -0.014601 |
| 37 | N14 - H30 | +0.007468 | +0.044417 | +0.063516 | -0.034442 |
| 38 | H34 - F41 | +0.008256 | +0.045569 | +0.378950 | -0.008846 |
| 39 | N16 - H31 | +0.196918 | -0.620050 | +0.038889 | -0.592902 |
| 40 | C19 - H32 | +0.283426 | -0.963798 | +0.035267 | -0.333838 |
| 41 | C22 - H33 | +0.287877 | -1.005572 | +0.014554 | -0.336469 |
| 42 | N14 - F41 | +0.007310 | +0.061213 | +2.299869 | -0.014094 |
| 43 | O36 - C37 | +0.252323 | -0.396669 | +0.018142 | -0.635893 |
| 44 | C37 - H39 | +0.284059 | -0.994620 | +0.038186 | -0.326648 |
| 45 | C37 - H38 | +0.277642 | -0.956950 | +0.041830 | -0.313105 |
| 46 | C37 - H40 | +0.277492 | -0.955425 | +0.041923 | -0.312872 |
| 47 | H30 - F41 | +0.020777 | -0.213906 | +0.003783 | -0.400776 |
